# Nanoscintillator-Mediated X-Ray Induced Photodynamic Therapy for Deep-Seated Tumors: From Concept to Biomedical Applications

**DOI:** 10.7150/thno.41578

**Published:** 2020-01-01

**Authors:** Wenjing Sun, Zijian Zhou, Guillem Pratx, Xiaoyuan Chen, Hongmin Chen

**Affiliations:** 1 State Key Laboratory of Molecular Vaccinology and Molecular Diagnostics & Center for Molecular Imaging and Translational Medicine, School of Public Health, Xiamen University, Xiamen 361102, China; 2 Laboratory of Molecular Imaging and Nanomedicine, National Institute of Biomedical Imaging and Bioengineering, National Institutes of Health, Bethesda, Maryland 20892, United States; 3 Department of Radiation Oncology, Stanford University School of Medicine, Stanford, California 94305, United States

**Keywords:** radiation therapy, X-ray excited optical luminescence, X-ray induced photodynamic therapy, nanosensitizers, deep-seated tumors

## Abstract

Photodynamic therapy (PDT) has shown great effectiveness in oncotherapy but has not been implemented in broad clinical applications because the limited penetration depth of the light used has been unable to reach deep-seated tumors. However, X-rays have been widely used in the clinical field for imaging and radiation therapy due to their excellent tissue penetration depth. Recently, X-rays have been established as an ideal excitation source for PDT, which holds great promise for breaking the depth limitation of traditional PDT for treatment of deep-seated tumors. This review aims to provide an overview of nanoscintillator-mediated X-ray induced PDT (X-PDT) including the concept, the design considerations of nanosensitizers for X-PDT, the modelling of nanosensitizer energy deposition, the putative mechanism by which X-PDT kills cells, and the prospects of future directions. We attempt to summarize the main developments that have occurred over the past decades. Possibilities and challenges for the clinical translation of X-PDT are also discussed.

## Introduction

Cancer is one of the world's most devastating diseases to the human population. Surgery, chemotherapy, and radiotherapy (RT) are the leading cancer treatment approaches in the clinical field, addressing the needs of almost all cancer patients 1. Although X-rays can fatally damage tumor cells, they also have a high toxicity to normal tissue 2. Hence, it is very important to strike a balance between inhibiting tumor growth and reducing side effects to normal tissue by controlling the dose of radiation administered to patients.

With the development of nanotechnology and nanomedicine, new strategies for cancer management have been developed. Photodynamic therapy (PDT) has been extensively applied as a less invasive, low toxicity, and highly selective therapeutic modality for clinical cancer treatment 3. Typically, in the presence of oxygen, photosensitizers are activated by light, of an appropriate wavelength, to produce cytotoxic reactive oxygen species (ROS) 3. Compared with chemotherapy and RT, PDT has an intrinsic safety and has fewer side effects to normal tissue. Breakthroughs in light-based diagnostic and therapeutic interventions have ushered in a new frontier in biomedical research. However, visible light suffers from poor transmission through tissues due to reflection, scattering, and absorption. The rapid decay in the intensity of the excitation light source as the light travels through tissues reduces the efficiency of tumor destruction when the tumors are in deep tissue. Achieving effective treatment of deep-seated tumors is still a major challenge for light-based diagnostics and therapeutics.

As an ionizing radiation with photon energies of kiloelectronvolts (keV) to megaelectronvolts (MeV) and excellent tissue penetration depth, RT is the leading cancer treatment approach, which addresses the needs of more than 70% of cancer patients. Ionizing radiation can induce DNA damage in cancer cells, resulting in highly efficient cancer cell destruction 4. However, to efficiently destroy cancer cells and inhibit tumor growth, a high dose of X-rays (50-80 Gy) is generally needed, especially for the treatment of deep-seated tumors, increasing the toxicity of radiation on the healthy tissue that lies close to the target tissue.

**Figure 1:**
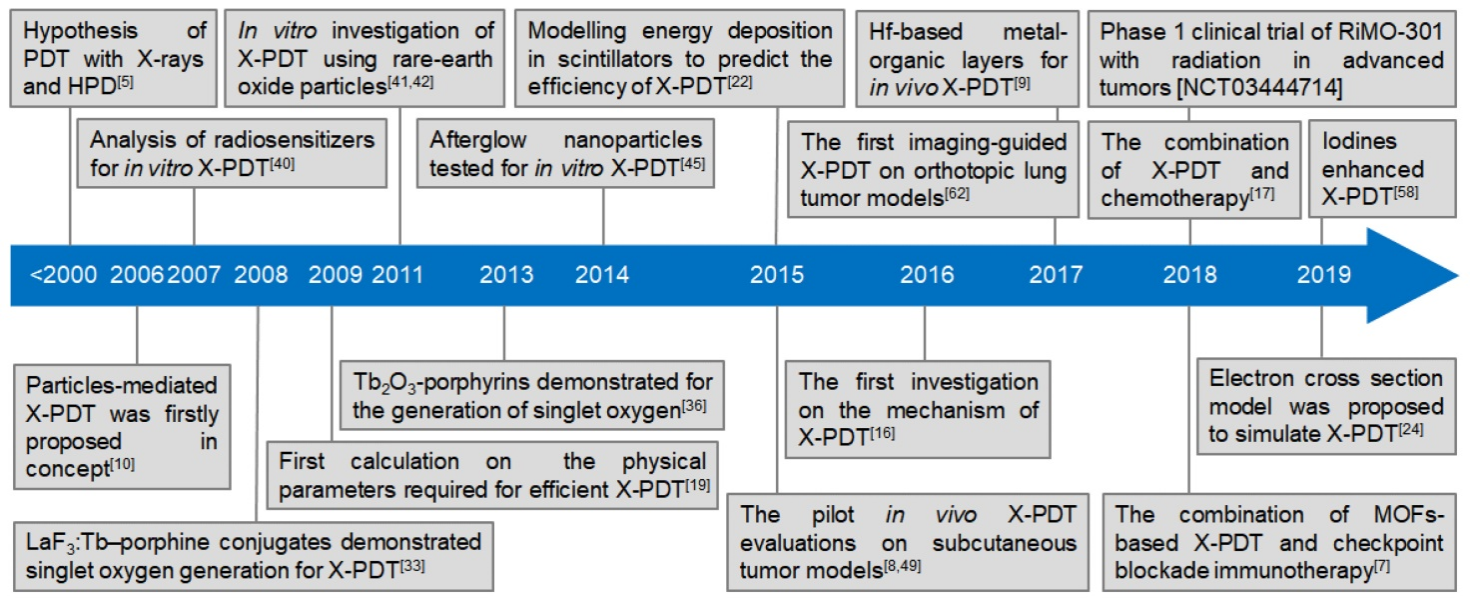
Timeline of the milestone research studies on X-PDT.

Researchers have thus hypothesized the use of PDT with X-rays, instead of laser light, and haematoporphyrin derivatives (HPD) to extend the possibilities of PDT 5. With the high tissue-penetrability of X-rays, researchers proposed X-ray induced photodynamic therapy (X-PDT) for improved therapeutic outcomes and reduced radiation damage to normal tissue for the treatment of deep-seated cancer. The principle of X-PDT is to use an energy transducer to transfer X-rays to optical luminescence and initiate the RT and PDT processes 6-9. Since the concept of nanoparticle-mediated X-PDT was proposed in 2006 10, X-PDT has undergone more than a decade of development *in vitro* and *in vivo* (Figure 1, Table 1), and several reviews focusing on the development of nanosensitizers have been published and references cited therein 11-13. A recent review has systematically described the interaction mechanisms between X-rays and X-ray-sensitive materials 14. Herein, this tutorial review aims to provide an overview of X-PDT, including the concept, the design considerations of nanosensitizers for X-PDT, the modelling of energy deposition in nanosensitizers, a possible cell-death mechanism initiated by X-PDT, and the prospects for future development. We attempt to summarize the main developments that have occurred over the past decades. Finally, possibilities and challenges for the clinical translation of X-PDT are also discussed.

## Principle of X-PDT

### X-PDT process

The energy of X-rays used in clinical RT is in the range of hundreds of keV to MeV. As a result, most traditional photosensitizers used for cancer PDT cannot be effectively activated by X-rays. In this regard, a physical transducer is required to absorb the X-ray irradiation energy and transfer it to photosensitizers to produce the cytotoxic singlet oxygen (^1^O_2_) necessary for tumor destruction. In the classical X-PDT model, this energy transfer is achieved by converting the absorbed x-ray energy it into optical photons of the appropriate wavelength that can be absorbed effectively by photosensitizers. These transducers are generally called scintillators and exhibit X-ray excited optical luminescence (XEOL). In addition, there are other possible mechanisms of energy transfer between X-ray absorbers and photosensitizers. For instance, acridine orange is a powerful photosensitizer, that has been shown in cancer models and sarcoma patients to be effective under low-dose X-ray irradiation, without use of a specific scintillator transducer 15.

**Figure 2:**
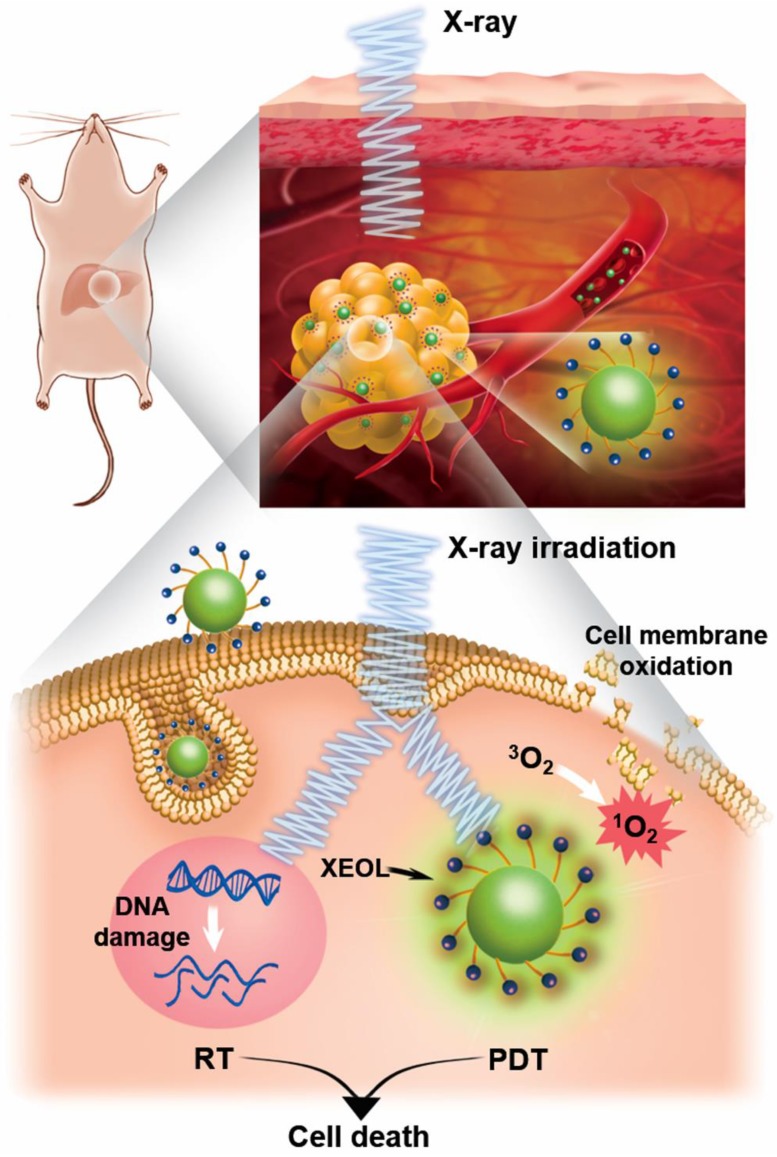
Schematic illustration showing the mechanism of X-PDT.

As illustrated in Figure 2, the classical X-PDT process can be divided into three main parts: (1) The nanoscintillators are irradiated by X-rays to generate XEOL. (2) The generated XEOL is absorbed by nearby, well-matched photosensitizers to produce ^1^O_2_, which can directly damage the cell membrane phospholipids of tumors while, at the same time, the absorbed ionizing radiation can generate radical species and break DNA double-strands. (3) The generated ROS induce cancer cell death by a combination of the PDT and RT processes to achieve effective cancer treatment. In this way, based on the effective energy transfer in the photosensitizer-loaded nanoscintillators (termed as nanosensitizers), X-rays can be used as the excitation light source to trigger PDT for the treatment of deep-seated tumors.

**Figure 3:**
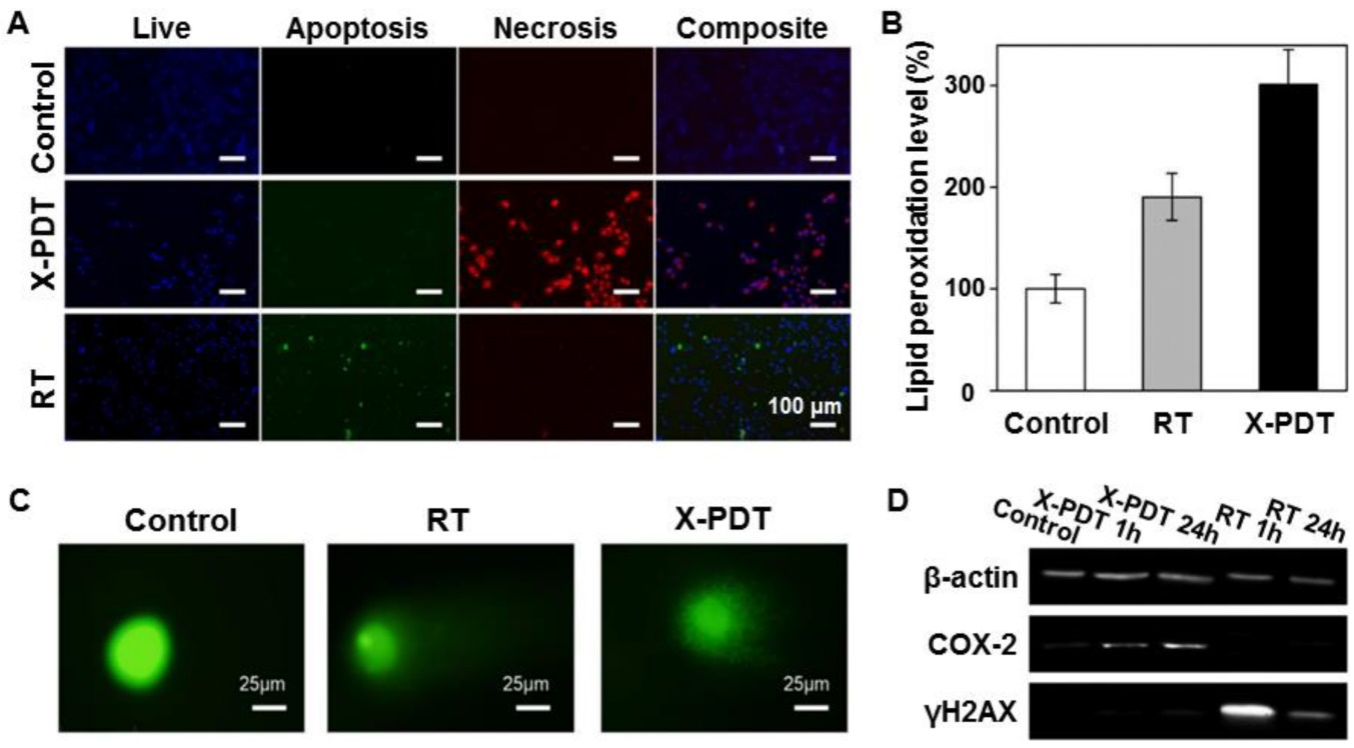
Investigation of the cell-killing mechanism of X-PDT. (A) Apoptosis and necrosis assay performed 24 h after X-PDT. (B) Lipid peroxidation assay. (C) Comet assay. (D) Western blot assay. H1299, a radioresistant non-small cell lung cancer cell line was employed. Adapted with permission from Ref 16. Copyright 2016 Ivyspring Publisher.

On account of the distinctive routes to cell death, each part in X-PDT suppresses the cell repair mechanism of the other, leading to enhanced treatment outcomes. As shown in Figure 3 16, compared with RT (0-5 Gy), X-PDT induced significant cell death and reduced clonogenicity at all doses. An apoptotic/necrotic assay (Figure 3A) indicated that cells treated with RT showed a modest level of apoptosis and no detectable necrosis at 24 h, while cells treated with X-PDT manifested extensive cell necrosis. These results demonstrated that a membrane-targeted PDT process could cause oxidative degradation of unsaturated lipids and surface proteins. A lipid peroxidation assay confirmed that both X-PDT and RT showed significant lipid oxidation, while X-PDT showed a 1.5-fold higher level of lipid peroxidation than that of RT only (Figure 3B). Also, the clear, comet-like appearance of cells and loosened nuclei indicated that both X-PDT and RT induced noticeable DNA damage (Figure 3C). A western blot assay further confirmed the impacts. As shown in Figure 3D, the levels of COX-2 (cyclooxygenase-2) and γ-H2AX (phosphorylated histone H2AX) both increased after the X-PDT treatment, caused by membrane oxidation and breakage of the DNA double-strand, respectively. These findings suggest that X-PDT combines the processes of RT and PDT. Recent studies further confirmed this mechanism for X-PDT 7, 17.

Experiments at the cellular level demonstrated that X-PDT is not only a PDT derivative, but also a type of RT derivative. PDT can excite photosensitizers to generate ^1^O_2_. RT can rapidly induce water radiolysis to produce hydroxyl radicals (•OH). The combination of RT and PDT, *i.e.* X-PDT, can produce a large amount of ROS, which plays an important role in causing damage to the cell membrane and DNA 18. The X-PDT strategy affords several benefits over conventional RT. First, X-PDT can kill cells that are resistant to RT alone (*e.g.*, glioblastoma cells, prostate cancer cells, and colorectal cancer cells). The PDT induced cell membrane damage, together with the radiation caused DNA damage, synchronously induce tumor cell apoptosis and necrosis. Second, the low irradiation dose (typical total dosage: <5 Gy) used in X-PDT is much lower than that of traditional clinical RT (typical total dosage: 60-80 Gy). Thus, X-PDT would have less side effects on normal tissue. Third, the X-PDT process can be activated with a single dose, at a lower dose rate compared with traditional RT. It is known that irradiation induced toxicity is positively correlated with the dose rate. In X-PDT, a typical irradiation dose necessary for treatment is in the range of 2-5 Gy, which is often comparable to one portion of the total dose used in conventional RT (*e.g.* 50-80 Gy in 2-Gy quantities).

### Design considerations of X-PDT

Despite the potential advantages of X-PDT over PDT, some crucial criteria need to be addressed before biomedical applications of the X-PDT strategy are possible. These criteria may enable more effective tumor ablation through the use of a nanosensitizer-mediated PDT process with low-dose X-rays.

**Figure 4:**
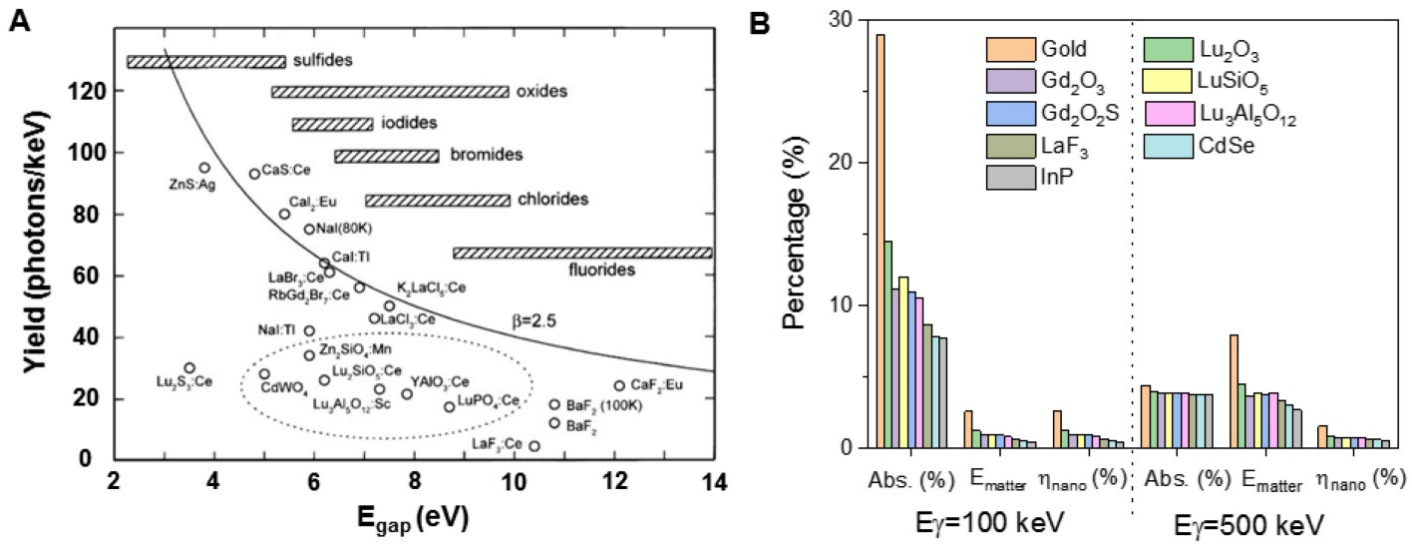
Theory simulation of a scintillator's effect on X-PDT. (A) Light yield of scintillators and cathode ray tube phosphors. Adapted with permission from Ref 21. Copyright 2002 Elsevier. (B) Energy deposited in matter (E_matter_) for one *γ* photon interaction in a tumor of volume *V*_tum_ loaded with 10 nm diameter nanoparticles, with an occupation ratio of 7 × 10*^-^*^3^. The absorption of the photons (Abs.) is calculated for the two considered energies (100 keV and 500 keV). *η*_nano_ quantifies the fraction of energy that is deposited in the nanoparticles themselves. Adapted with permission from Ref 22. Copyright 2015 Royal Society of Chemistry.

Since the initial proof-of-principle study of X-PDT, several theoretical models have been generated to guide the development of nanosensitizers and estimate the efficacy of X-PDT. The initial simulations put forth by Morgan and coworkers, however, were not encouraging 19. Morgan *et al.* estimated the X-ray energy and dose required to produce a lethal dose of ^1^O_2_ in tissue, depending on the radiation dose absorption efficiency of the nanoscintillators and the ^1^O_2_ generation after absorption of X rays (per MeV). Therefore, based on the reported lethal dose of ^1^O_2_, the authors gave a practical relationship between the necessary emission yield of nanoscintillators (photons·MeV^-1^) and the incident X-ray energy for estimating the feasibility of new, candidate nanoscintillator-photosensitizer conjugates for use in X-PDT. Based on the assumption that X-ray photons which hit a nanoparticle would transfer all of their energy to that nanoparticle, the study concluded that the strategy would be effective for photon sources at intensities <300 kV due to enhanced X-ray absorption near the K-edge of the nanoparticle material 20. However, many uncertainties existed in the simulation, including large variations in the reported values for cellular uptake and the singlet oxygen lethal dose, wildly optimistic assumptions, and oversight of physical radiosensitization effects associated with the presence in cells of high-Z X-PDT nanoscintillators. If and how the time scale for delivery of singlet oxygen affects the dose required for cell death were not addressed. Additionally, Morgan only showed the simulated results using LaF_3_ nanoparticles, which have a low light yield under X-ray irradiation (Figure 4A). Overall, this simulation was a first attempt and raised valid questions about the practicality of X-PDT as an effective treatment option. Figure 4A shows the light yield limits for several commonly used scintillators in X-ray imaging screens and films for projection imaging, fluoroscopy, and computed tomography (CT) 21. As indicated, the fundamental limit is the smallest for fluorides, which have the largest band gap, and the largest for sulfides, which have the smallest band gaps.

Bulin *et al.* found that Morgan's simulation was restrictive and overestimated the energy deposition in nanoscintillators 22. An alternative model for nanoscintillator-mediated PDT was proposed to better simulate of the efficiency of X-PDT using a GEANT4-based Monte Carlo program 22. This model takes into account that secondary electrons generated in the nanoscintillator have ranges much greater than the size of the nanoscintillator and thus most of the X-ray energy is transferred to the medium outside the nanoscintillator. The conditions in Bulin's simulation were restricted to 10 nm diameter nanoparticles distributed in a tumor with a high occupancy ratio, and the spatial energy distribution that resulted from the interaction between the X-ray photons and the nanosensitizers was estimated. In this simulation, a new loss parameter η_nano_, which corresponds to the fraction of total energy that is deposited in the scintillators in the mixed media, was introduced. Using this parameter, the study finds that, most of the X-ray energy is absorbed by water molecules and does not contribute to scintillation. The process of excitation of nanoscintillators by X-rays can therefore be broken down into two phases. In the first phase, X-ray photons are absorbed by the medium and transfer their energy to electrons. Due to its higher abundance, water is the primary absorber of X-ray radiation. In the second phase, these energetic electrons propagate through the tissue and deposit energy along their path through inelastic scattering. When an electron passes through a nanoparticle, some of its energy can be transferred to stimulate the emission of scintillation photons. The amount of energy transferred is a function of the electron's energy, the nanoparticle size and its material composition (electron stopping power and density). This indirect transfer of energy is the primary mechanism for X-ray luminescence of nanoscintillator suspensions. Considering the linear energy transfer of electrons, the typical energy transfer between an electron and a nanoparticle with a diameter of 20 nm is on the order of 10 eV. Based on the simulation, the calculations for various nanomaterials under 100 and 500 keV irradiations are listed in Figure 4B. As indicated, gold has the largest absorption capacity for X-ray photons (Abs.) of all the materials listed and its use results in the highest energy deposition in matter (E_matter_) 23. However, only a small percentage of the energy deposition is transferred to nanoparticle, as quantified by the loss parameter η_nano_.

As previously discussed, at physiological nanoparticle concentrations (i.e. <1 mg·ml^-1^), the absorption of energy by nanoparticles is mainly driven by inelastic electron scattering and is nearly independent of the nanoparticle X-ray stopping power. Following this, Klein et al proposed a simplified model based on electron cross sections to describe this process 24. The X-ray luminescence yield is a function of the radiation dose to the tissue (Gy=J·kg^-1^), the nanoparticle mass concentration *C_NP_* (g·cm^-3^), the light yield of scintillator (*Y_sc_*), and the electron cross-sections (*μ/ρ*) of the tissue and the nanoparticle material. Using this framework, the density of photons emitted by a dilute suspension of scintillators (*N_scint_*) can be computed according to:


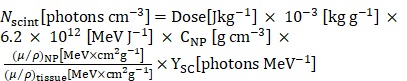
 (1)

(where *μ/ρ* can be obtained from the ESTAR database of the National Institute of Standards and Technologies (NIST)).

The model further assumed that all photons were converted to singlet oxygen, and cells are spherical structures with a diameter of 10 μm. Accordingly, the number of singlet oxygen per cell (*N*_1O2_) was calculated as:


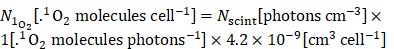
 (2)

Compared to to the X-ray cross-section, the electron absorption cross-section is a better predictor of the efficacy of X-PDT, at least within the correct order of magnitude, as indicated by the sample photon yields that were computed. In fact, it has been shown that the model provides an upper bound for the actual number of scintillation photons emitted. Using the same assumption and LaF_3_ as the sample, the electron cross-section model predicts ~10^6 1^O_2_ molecules per cell over a wide energy range of excitation energies, which is below the required 10^7 1^O_2_ per cell PDT threshold for cell killing. It should be noted that this model does not account for the microscopic inhomogeneity in radiation dose that results from the high density of nanoparticles compared to water. This local increase in dose and ROS production around dense nanoparticles is being investigated as a potential approach for increasing the efficacy of cancer radiotherapy. Most nanoscintillators are made of dense materials, and therefore they also result in local radiosensitization independently of PDT. For greater accuracy, Monte Carlo simulations, which model photon-nanoscintillator interactions in greater detail, are necessary.

Taken as a whole, the classical model of X-PDT is unable to fully explain the efficacy of X-PDT regimens seen during pre-clinical tests. The luminescence of nanoscintillator suspensions excited by X-ray is several orders of magnitude lower than the optical photon dose required for conventional PDT. The efficacy of X-PDT may therefore be due to synergistic effects between RT and PDT 25. The X-PDT process may also involve non-optical forms of energy transfer between nanoparticles and covalently bound photosensitizers, such as Förster resonance energy transfer (FRET) and low-energy Auger electrons. Last, an often-ignored side-effect of X-PDT is that the presence of high-Z nanoparticles within cells enhances radiation dose within nanometers of their surface 26. This effect can increase cell killing regardless of PDT, thus suitable controls must be employed when testing X-PDT agents. Nanoparticles can also catalyze the radiation-induced formation of radicals and ROS, further enhancing cell killing 27. Thus, further work remains to be done to fully elucidate the chain of physicochemical events that enables X-ray radiation to activate photosensitizers.

**Figure 5:**
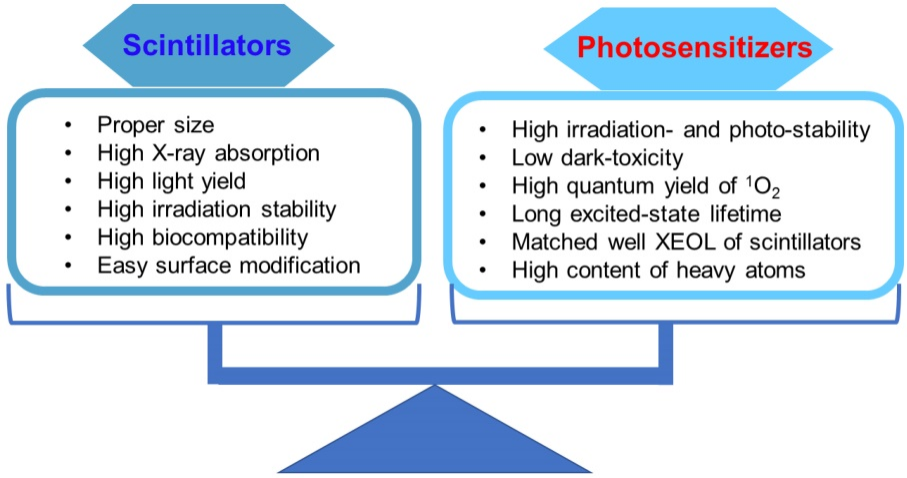
Optimization of both scintillators and photosensitizers to achieve efficient and enhanced X-PDT.

The results derived from these simulations provide important guidelines for the design of nanosensitizers and for quantifying ^1^O_2_ generation in the frame of therapy which combines RT and the PDT effects. However, many factors, especially scintillation light yield, biodistribution after injection, photosensitizers, efficiency of transfer toward photosensitizers, and efficiency of irradiation absorption of radiosensitizers, will affect the efficiency of X-PDT. As shown in Figure 5, the commonly considered characteristics of scintillators and photosensitizers when designing nanosensitizers for X-PDT are listed. First, to effectively trigger PDT under X-ray irradiation, the scintillators should have a strong absorption capacity for irradiation, and strong X-ray-excited optical luminescence (>15 photons·keV^-1^). Figure 4A reveals the light yields of several of the most commonly used scintillators, which showed that the light yield for an ideally activated scintillator would have a maximum energy efficiency of close to 40% 21. Although there may be a limitation on the light yield, a recent study designed a new class of scintillators, which had strong X-ray absorption and XEOL 28. There, the Huang group prepared all-inorganic perovskite scintillators with size of 9.6 nm using a wet chemical synthesis approach. Compared with conventional bulk scintillators (CsI:Tl, PbWO_4_, YAlO_3_:Ce, and Bi_4_Ge_3_O_12_), the perovskite scintillators converted small doses of X-ray photons into multi-colored visible light. Furthermore, to achieve biomedical applications, the as-prepared scintillators are required to have good biocompatibility, such as low-toxicity, weak cellular immune response, minimal protein adsorption and cell adhesion, long blood circulation, and great capabilities for escaping the reticuloendothelial system. Besides, the scintillators of a suitable size generally benefited from passive tumor accumulation mediated by the enhanced permeability and retention (EPR) effect. It is important to note, however, that when the EPR effect was first discovered, it was extolled by many as a magic weapon against cancer; it is clear now that this is not the case. Delivering nanoparticles to cancer cells is an extremely complicated process. One of the major challenges in the field of tumor-targeting delivery is that the EPR effect is much more pronounced and uniform in xenograft models than it is in most human cancers. Reviews on this and other key problems have been carefully listed and discussed at length 29, 30. Second, photosensitizers should have excellent irradiation stability, high ^1^O_2_ quantum yield in aqueous solutions, and good biocompatibility. Third, to achieve highly efficient energy transfer from scintillators to photosensitizers, the absorption of the photosensitizers should have maximum overlap with the XEOL of the scintillators. Also, an appropriate distance between scintillators and photosensitizers is required for effective energy transfer. Last but not least, for *in vitro*/*in vivo* applications of X-PDT, the as-prepared scintillators should be modified with a proper surface coating and conjugated with tumor targeting ligands. Such modifications enable nanosensitizers to have good performance in biological systems, such as improved pharmacokinetics, high selectivity to cancer cells, preferential accumulation in tumor tissue, and rapid clearance from normal tissue 31. In all, these optimization strategies will give rise to efficient tumor accumulation and destruction, and reduce damage to normal tissues as well. Researchers in this area have made great efforts to balance the properties of scintillators and photosensitizers. In the next section, we will introduce the typical scintillators and matched photosensitizers to evaluate X-PDT efficacy. These scintillators include fluorides, oxides, micelles, high-Z organometallic complexes, phosphors, metal-organic complex containing heavy metal clusters, and luminescent organic bridging ligands.

## Current development of classic X-PDT

As described above, the concept of nanoparticle-mediated X-PDT was first proposed in 2006 10. Since then, there have been many attempts to advance the development of X-PDT. Figure 1 presents a timeline of representative milestones in X-PDT technology. Table 1 gives a more detailed summary of these findings, which can be separated into three developmental stages. During the initial period, research demonstrated that X-PDT can generate ^1^O_2_ in solution using fluorides and oxides as the transducers at the same dosages used for RT (*i.e.* 6-8 Gy). Then, the investigations focused on *in vitro* demonstrations under a selected dosage for RT. There was no demonstration of this technology *in vivo* using a low-dosage of X-rays until 2015. Progress in the design and modification of nanosensitizers has led to more comprehensive studies. In the following section, we will give examples and discuss the classic X-PDT scheme based on the time lines in Figure 1.

### Early investigations of X-PDT in solution

**Figure 6:**
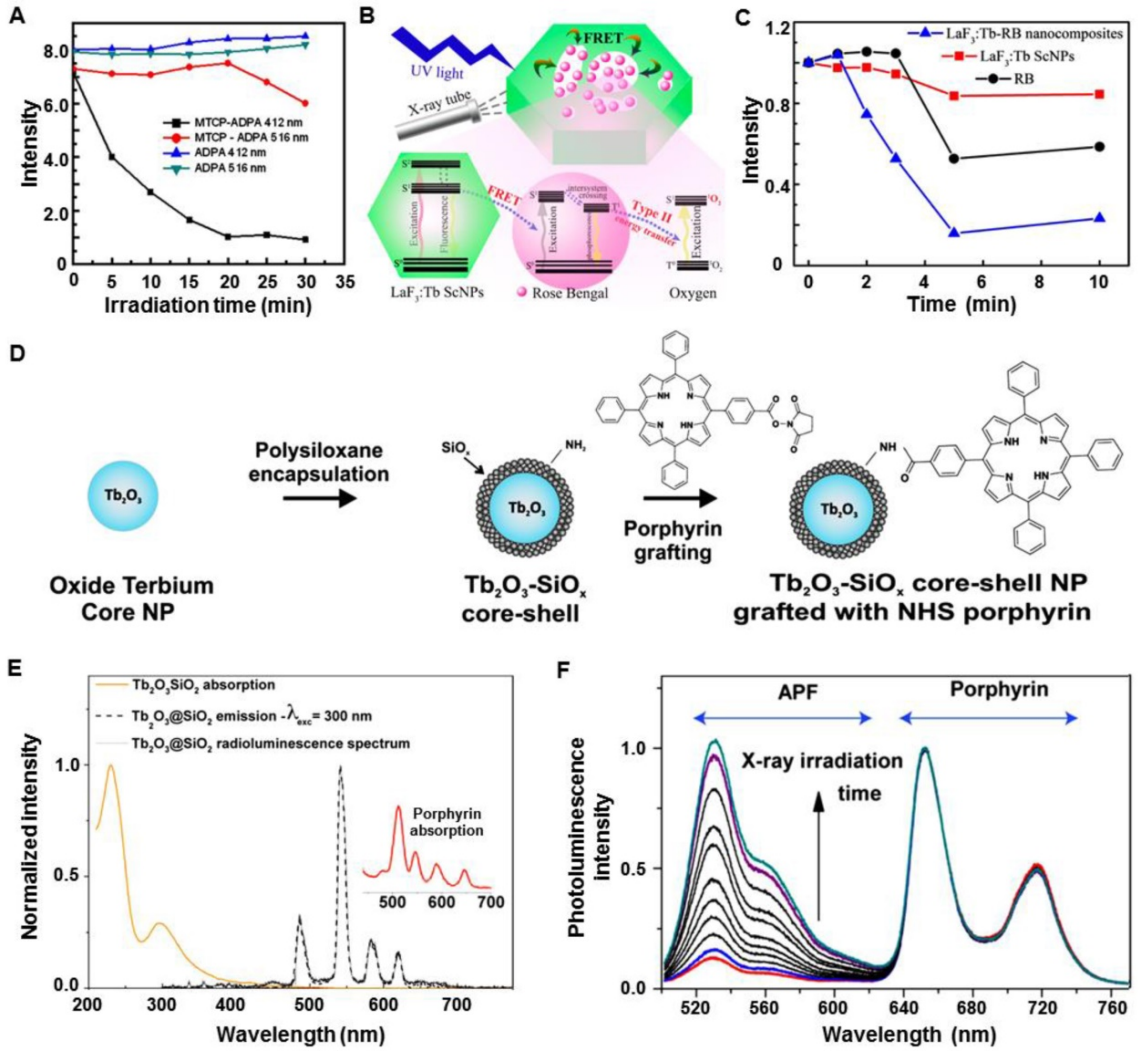
Fluorides and oxides for use with of X-PDT. (A) Quenching of ADPA with X-ray irradiation. Adapted with permission from Ref 33. Copyright 2008 American Institute of Physics. (B) Scheme of LaF_3_:Tb-RB-mediated X-PDT. (C) Decrease in the emission of DPBF treated with LaF_3_:Tb-RB and X-rays. Adapted with permission from Ref 34. Copyright 2015 American Chemical Society. (D) Synthesis of porphyrin-Tb_2_O_3_@SiO_2_. (E) XEOL of Tb_2_O_3_@SiO_2_. (F) ^1^O_2_ generation of porphyrin-grafted Tb_2_O_3_@SiO_2_ under X-ray irradiation. Adapted with permission from Ref 36. Copyright 2013 American Chemical Society.

In the early stages, most of the investigations demonstrated the feasibility of ^1^O_2_ generation by employing fluorides and oxides of scintillators as transducers. As one of the mostly studied scintillators, rare-earth-element doped nanomaterials can absorb incoming high energy radiations and transfer them to luminescent centers, resulting in efficient luminescence in the visible light region 32. Porphyrin is a photosensitizer that is commonly applied in clinical PDT, and Tb^3+^ exhibits efficient green luminescence that matches well with the absorption band of porphyrin. In 2008, the Chen group synthesized meso-tetra(4-carboxyphenyl) porphine (MTCP) conjugated LaF_3_:Tb nanoparticles (LaF_3_:Tb-MTCP) to investigate ^1^O_2_ generation following the X-PDT process 33. The nanoparticles were about 10-15 nm in size, which emitted green XEOL centered at 540 nm. Under X-ray irradiation (0.44 Gy·min^-1^, 13.2 Gy), the luminescence of 9,10-anthracenedipropionic acid (ADPA) was quenched by both MTCP and LaF_3_:Tb-MTCP, with the quenching by the latter more than doubled (Figure 6A). In another study, the Yang group demonstrated X-PDT effects using mesoporous LaF_3_:Tb nanoparticles (Figure 6B) 34. Under X-ray irradiation, the green XEOL of LaF_3_:Tb could be absorbed by an adsorbed rose bengal (RB) (Figure 6B). Under X-ray irradiation, RB alone could quench about 40% of the fluorescence of 1,3-Diphenylisobenzofuran (DPBF), while the quenching induced by RB-adsorbed LaF_3_:Tb was about two-fold higher than that of RB alone (Figure 6C). Then, the same group coated a layer of silica on the mesoporous LaF_3_:Tb scintillators 35. After integration with RB, the nanosensitizers showed an efficient generation of ^1^O_2_ under X-ray irradiation. Besides fluorides, oxides were also employed to investigate the efficacy of X-PDT. The Dujardin group conjugated silica coated Tb_2_O_3_ (Tb_2_O_3_@SiO_2_) core-shell nanoparticles with porphyrin (Figure 6D) 36. The XEOL matched well with the absorption of porphyrin (Figure 6E), and ^1^O_2_ was generated under X-ray irradiation (5.4 mGy·s^-1^), as indicated by an increase in the emission of 3-p-(amino phenyl) fluorescein (APF) (Figure 6F).

**Figure 7:**
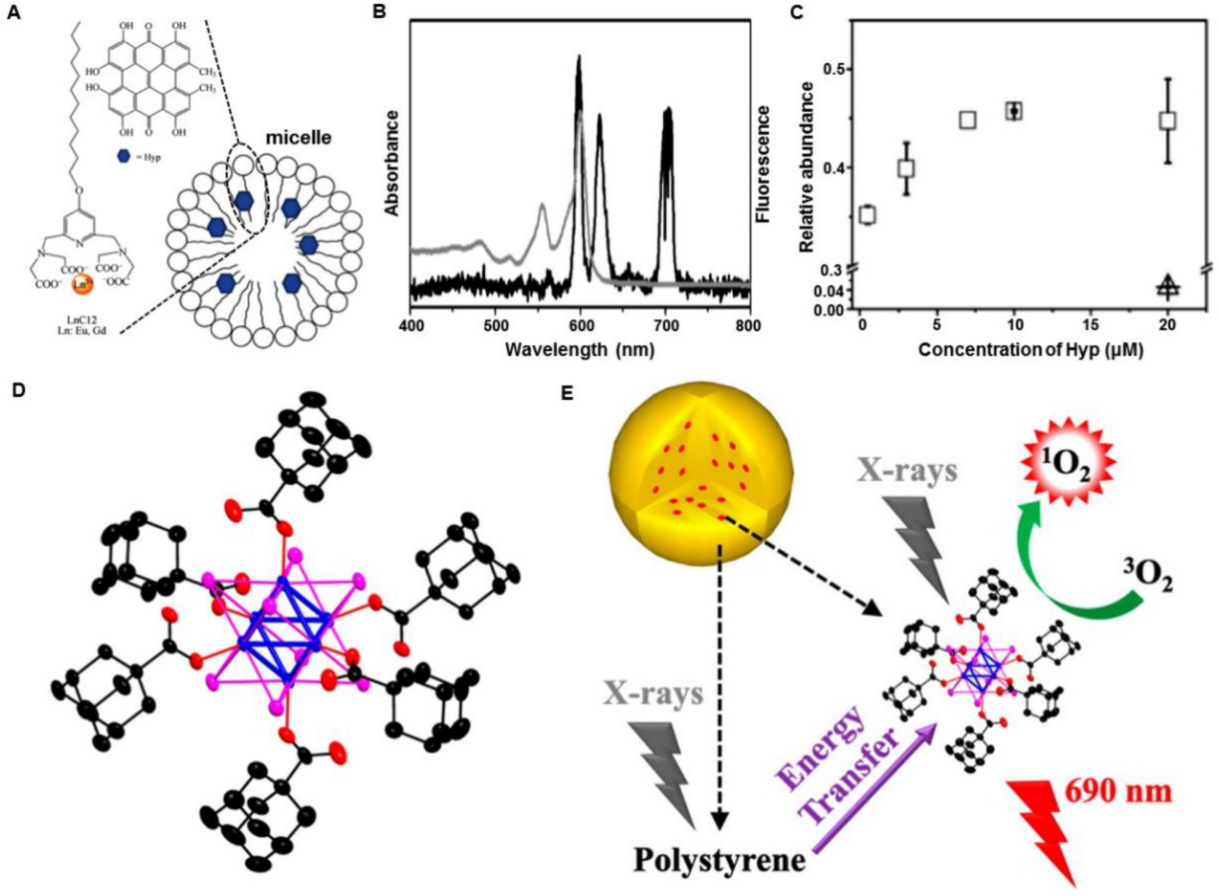
(A) Schematic representation of the Hyp-GdEuC1_2_ micelles and the respective structures of the amphiphilic GdLnC1_2_ complex and the Hyp. (B) Overlay between the XEOL of EuCl_3_ (black line) and the absorption spectrum of Hyp (gray line). (C) ^1^O_2_ production of Hyp-GdEuC12 micelles as indicated by the abundance of 1-pyrenecarboxaldehyde, measured by mass spectrometry (∆: non-irradiated, □: irradiated). Adapted with permission from Ref 37. Copyright 2015 Springer. (D) Crystallographic structure of (n-Bu_4_N)_2_[Mo_6_I_8_(OOC-1-adamantane)_6_] (blue, molybdenum; magenta, iodine; red, oxygen; black, carbon; hydrogen atoms are omitted for clarity). (E) Scheme of (n-Bu_4_N)_2_[Mo_6_I_8_(OOC-1-adamantane)_6_] cluster-mediated X-PDT. Adapted with permission from Ref 39. Copyright 2015 American Chemical Society.

Other lanthanide-based structures were also investigated. For example, the Réfrégiers group reported a hydrophilic micelle for X-PDT 37. The micelles consisted of lanthanide chelates GdEuC1_2_ and hypericin (Hyp) as photosensitizers that were incorporated into their hydrophobic regions (Figure 7A). Upon X-ray irradiation, the GdEuC1_2_ micelles showed characteristic luminescence of Eu^3+^ in the visible spectral region, which matched well with the absorption of Hyp (Figure 7B). The ^1^O_2_ production was illustrated by an abundance of 1-pyrenecarboxaldehyde, which was measured by mass spectrometry (Figure 7C). As a high-Z organometallic complex, [M_6_Li_8_La_6_]_n_ (M = Mo, W, Re) clusters were reported to be exclusively quenched by molecular oxygen to form ^1^O_2_ 38. Octahedral molybdenum (n-Bu_4_N)_2_[Mo_6_I_8_(OOC-1-adamantane)_6_] clusters were embedded in polystyrene films to form an aggregate and enhance radioluminescence (Figure 7D) 39. Obvious characteristic emission at 1274 nm was observed, indicating ^1^O_2_ generation. Based on these results, the mechanism of cluster-mediated X-PDT was proposed (Figure 7E). Following the concept of nanoparticle-mediated X-PDT, these attempts made in the early stages have propelled the development of feasible methods for the design of nanosensitizers and their applications in cancer management.

### X-PDT for killing cancer cells

The above studies have demonstrated the ability of the X-PDT strategy to produce ROS in solution. This strategy was then applied to killing cancer cells, where the effective cellular uptake should first be considered in order to generate cytotoxic ROS intracellularly.

Following the concept of X-PDT, the Misawa group analyzed a number of radiosensitizers for X-PDT *in vitro* 40. The radiosensitizers, including TiO_2_, ZnS:Ag, CeF_3_, and quantum dots (CdTe and CdSe), in particulate form were designed to generate ROS inside or outside HeLa cells. Under high-doses of X-ray irradiation, a proportional increase of ROS generation was observed with increasing concentrations of TiO_2_, ZnS:Ag, CeF_3_, and CdSe quantum dots. Also, the survival fraction of the HeLa cells, obtained by a cell proliferation kit, showed the insignificant effects of the sensitizing materials, compared with the control group (*i.e.,* cells irradiated directly by X-ray). To enhance the sensitization effect, surface modification of the radiosensitizers was used to help internalize radiosensitizers into the HeLa cells and reduce the cell viability. However, the cell damage mechanism employed by these radiosensitizers was different from classical RT caused by double-strand breakage in DNA. Analysis of the cell damage mechanism by these radiosensitizers under X-ray irradiation needs further investigation.

**Figure 8:**
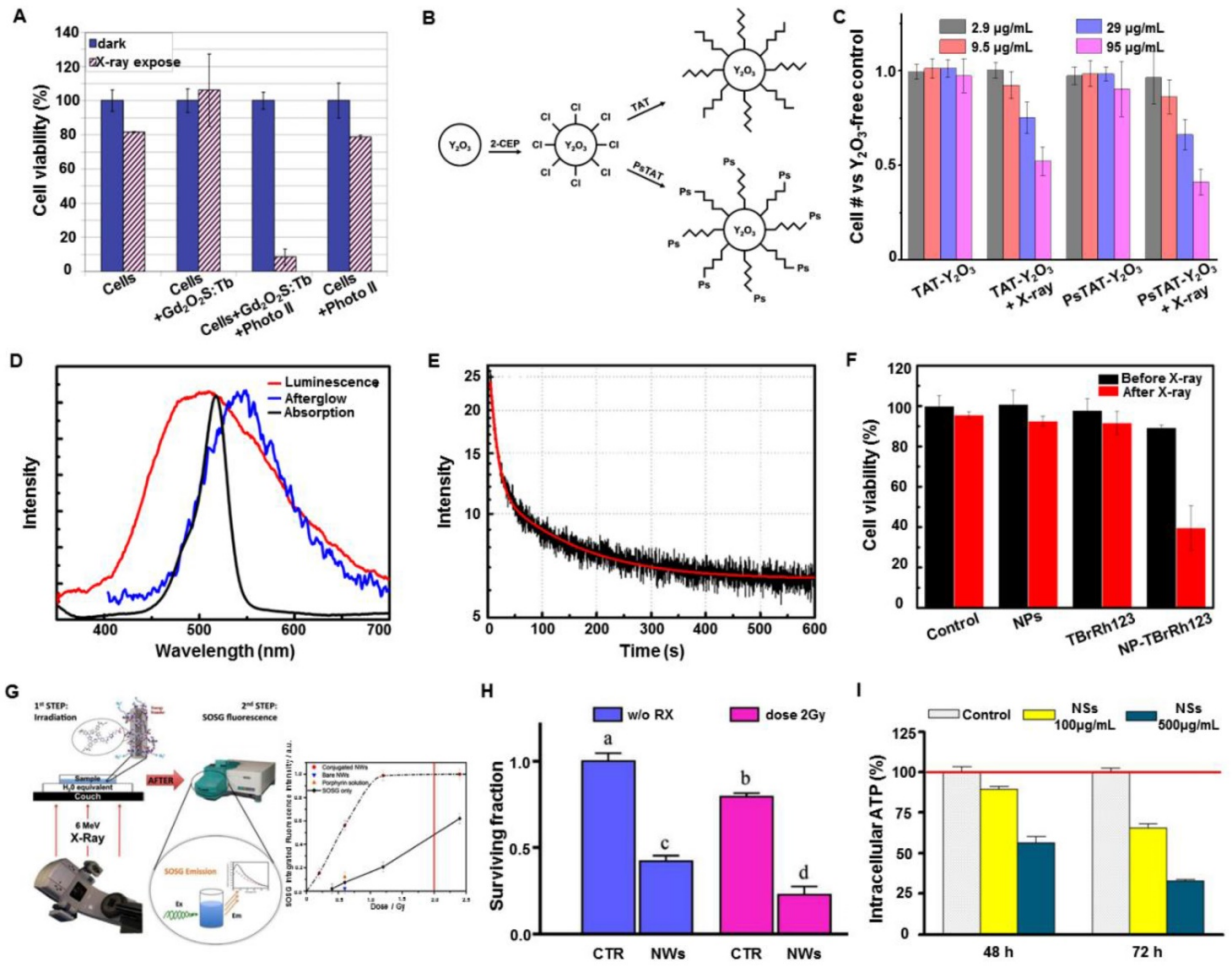
(A) Cellular metabolic activities using Gd_2_O_2_S:Tb particles as transducers under X-ray irradiation. Adapted with permission from Ref 41. Copyright 2011 IOS Press. (B) Synthesis of TAT- and PsTAT-Y_2_O_3_ scintillators. (C) Percentage of cancer cells remaining after the treatment with TAT-Y_2_O_3_ or PsTAT-Y_2_O_3_ under X-ray irradiation. Adapted with permission from Ref 42. Copyright 2011 American Chemical Society. (D) The X-ray excited luminescence and afterglow spectra of ZnS:Cu,Co scintillators overlapped with the absorption of TBrRh123. (E) Afterglow decay of ZnS:Cu,Co scintillators. (F) Viabilities of PC3 cells treated with ZnS:Cu,Co-TBrRh123 before and after X-ray irradiation. Adapted with permission from Ref 45. Copyright 2014 American Institute of Physics. (G) Scheme illustrating the ^1^O_2_ production from porphyrin-SiC/SiOx nanowires (NWs) excited by X-rays. (H) Surviving fraction of cells treated only with porphyrin-SiC/SiOx NWs (50 μg·mL^-1^), only with radiation (2 Gy), or a combination of porphyrin-SiC/SiOx NWs and X-rays (2 Gy). Each letter indicates a different level of significance (p<0.05). (I) ATP levels in A549 cells treated with porphyrin-SiC/SiOx and X-rays. Adapted with permission from Ref 46. Copyright 2015 Nature Publishing Group.

The Tata group employed terbium doped gadolinium oxysulfide particles (Gd_2_O_2_S:Tb, 20 micron dimension) to activate Photofrin II (Photo II) through X-ray induced luminescence, and evaluated the efficacy of X-PDT on the human glioblastoma cell line 41. Severe suppression in the cellular metabolic activity was observed under clinically relevant conditions (Photo II at 20 µg·mL^-1^) with Gd_2_O_2_S:Tb particles and X-rays (120 kVp) (Figure 8A). However, the micron-sized particles suffered from the poor circulation features in a living body, hampering the *in vivo* applications. To minimize the issues from size, the Vo-Dinh group functionalized commercial yttrium oxide (Y_2_O_3_) scintillators (~12 nm diameter) with a psoralen (Ps)-containing, thiol-modified fragment of the HIV-1 TAT peptide and evaluated the efficacy of *in vitro* therapy using X-PDT (Figure 8B,C) 42.

Persistent luminescence (also called afterglow) phosphors can emit luminescence long after excitation, and have been used as nanoprobes in small animal optical imaging without the autofluorescence background interference 43. Persistent luminescence allows the host to store the excitation energy, and then slowly release the energy from the trapped charge carriers to emit a long-lasting phosphorescence. This unique property can be used as a potential energy mediator for PDT treatment 44. The Chen group first employed ZnS:Cu,Co afterglow nanoparticles to investigate the efficacy of X-PDT *in vitro* 45. As first generation persistent luminescence materials, ZnS:Cu emits green luminescence, which overlaps well with the absorption of tetrabromorhodamine-123 (TBrRh123) (Figure 8D). Furthermore, the XEOL afterglow of ZnS:Cu,Co can last for more than 10 min after X-ray irradiation is ended (Figure 8E). The nanosensitizers were efficiently ingested by the cancer cells, inducing a sharp decrease of cell viability under X-ray irradiation (2 Gy). However, the applied X-ray irradiation alone had a negligible effect on the destruction of the cancer cells (Figure 8F). This study made afterglow nanoparticles a potential candidate as a light source for activating PDT for deep-seated tumors.

Besides transitional optical phosphors, other materials were also evaluated as nanosensitizers to achieve X-PDT. The Salviati group irradiated porphyrin conjugated SiC/SiOx nanowires using a clinical RT instrument with a relatively low X-ray irradiation dose (2 Gy, 6 MeV) (Figure 8G) 46. The cell population was reduced by about 75%, with respect to control cells, as evidenced from the clonogenic survival assay (Figure 8H). Further experiments showed a remarkable reduction of intracellular adenosine triphosphate (ATP), which is directly correlated with cell viability (Figure 8I). However, the mechanism of ^1^O_2_ generation in this research was unclear, as there was no evidence showing that the nanowires could emit XEOL under X-ray irradiation.

As investigated by the above evaluations in solution and at cellular levels, these results have proven that under X-ray irradiation, transducers can transfer irradiated energy to bound photosensitizers to generate ROS and induce cell death. Although some cases do not exactly follow the mechanistic cell death of X-PDT, we still can learn from these studies and gather some strategies for *in vivo* applications.

### *In vivo* X-PDT for cancer treatment

In the first 10 years of development, there were virtually no X-PDT studies performed *in vivo*. The proposer of the concept of X-PDT commented in 2014 that “previous attempts using X-rays to activate photosensitizers were not very successful, since the traditional PDT photosensitizers could not be efficiently activated by X-rays” 47. To bring X-PDT technology forward, it is critical to demonstrate that nanosensitizers can be excited by external X-ray irradiation to generate XEOL and activate X-PDT *in vivo*. In this section, we will summarize the progress of transforming the X-PDT concept into more realistic *in vivo* applications.

Besides commenting on previous investigations, the Chen group also exploited copper-cysteamine complex (Cu-Cy) microparticles (3-10 µm) and evaluated their X-PDT performance for *in vivo* cancer treatment 47. An *in vitro* study on human breast cancer cells (MCF-7) showed significant cell death using Cu-Cy particles activated by X-rays, and *in vivo* treatment that was conducted on a subcutaneous tumor model inhibited tumor growth after an intratumoral injection of Cu-Cy particles and X-ray irradiation (5 Gy). These results demonstrated that Cu-Cy particles could be efficiently activated by X-rays to produce ROS for cancer treatment. Although the results were positive, the exact mechanism of ROS production was not clear, as there was no photosensitizer in the structure of Cu-Cy. A recent study demonstrated the Cu-Cy nanoparticles could regulate the tumor microenvironment for *in situ* glutathione (GSH)-activated and H_2_O_2_-reinforced chemodynamic therapy for drug-resistant breast cancer 48. The Cu-Cy nanoparticles could generate toxic •OH *via* a Fenton-like reaction by reacting with local GSH and H_2_O_2_.

**Figure 9:**
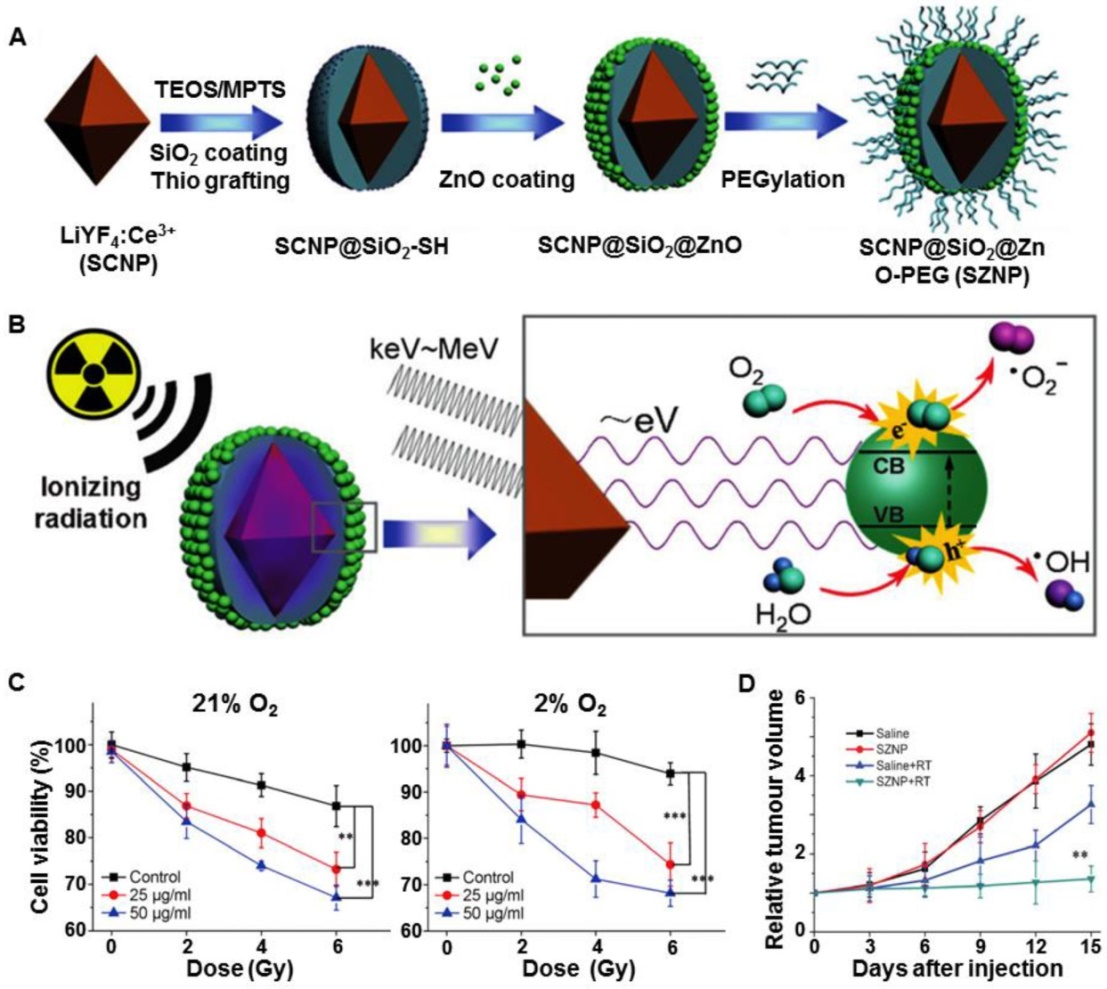
(A) Schematic illustration of the synthetic route to ZnO coated LiYF_4_:Ce (SZNP). (B) The mechanism of SZNP-mediated X-PDT. (C) Viabilities of normoxic and hypoxic HeLa cells treated with SZNPs for 24 h under X-ray radiation (0, 2, 4, 6 Gy). (D) *In vivo* evaluation of SZNPs-mediated synchronous RT and PDT. Adapted with permission from Ref 49. Copyright 2014 Wiley-VCH.

The efficacy of X-PDT *in vivo* was further demonstrated by others and us 8, 49. The Shi and Bu group coated a semiconductor layer of ZnO on Ce-doped LiYF_4_ nanoparticles (LiYF_4_@ZnO) and then investigated the *in vivo* efficacy of X-PDT with minimal oxygen dependence (Figure 9A) 49. Ce-doped LiYF_4_ exhibited strong emission bands in the Ultraviolet (UV) region (305 and 325 nm) that matched the bandgap of the surface-bound ZnO layer. The subsequently formed excitons (the electron-hole (e^-^-h^+^) pairs) interacted with water and oxygen molecules to form free radicals (Figure 9B). These ROS were mainly produced through an oxygen-independent PDT (type I reaction) mechanism, and could cause irreversible oxidative damage to DNA, lipids, and proteins, even in the low oxygen environment. *In vitro* studies showed significant ROS production under X-ray irradiation (3 Gy), regardless of the oxygen tension, confirming the highly efficient X-PDT both in normoxic (21% O_2_) and hypoxic (2% O_2_) cells (Figure 9C). Furthermore, the *in vivo* study showed that the tumor growth was significant inhibited after intratumoral injection of LiYF_4_@ZnO into subcutaneous tumors and irradiation with X-rays (8 Gy) (Figure 9D). Following this strategy, the Bu group further combined scintillators and heavy metals to absorb X-rays and transmit the energy to photosensitizers 17. In this design, LiLuF_4_:Ce generated X-ray excited UV luminescence, which was absorbed by the photosensitizers (Ag_3_PO_4_) to generate •OH. To make full use of the UV luminescence produced by LiLuF_4_:Ce, a cisplatin prodrug Pt(IV) was utilized as a sacrificial electron acceptor to increase the yield of •OH by separating the electrons and holes in Ag_3_PO_4_. Additionally, cisplatin was produced upon the reduction of Pt(IV) and further enhanced the damage caused by •OH. Through a two-step amplification strategy, LiLuF_4_:Ce@Ag_3_PO_4_@Pt(IV) nanoparticles (LAPNP) enhanced the curative effects of X-PDT.

**Figure 10:**
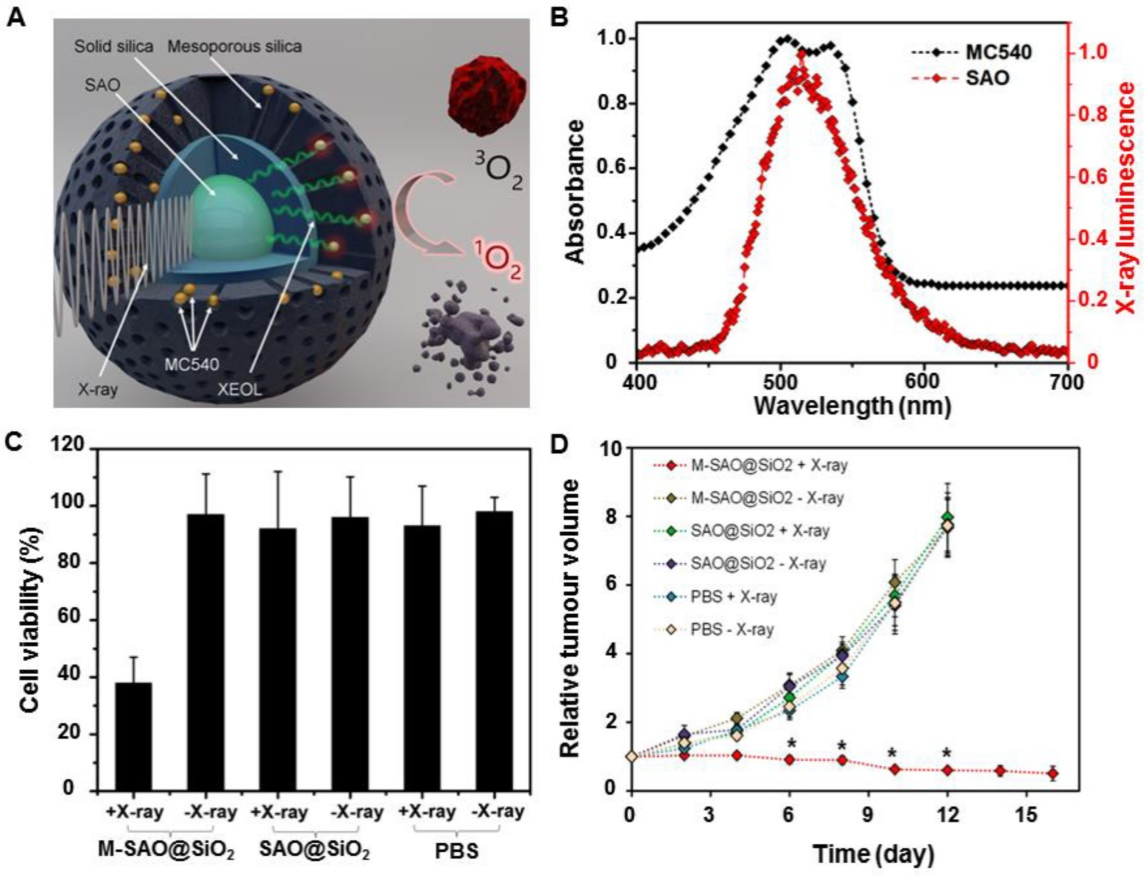
(A) Schematic illustration of the structure of M-SAO@SiO_2_ and the working mechanism of SAO-based X-PDT. (B) The XEOL of SAO (red) and the absorption of MC540 (black). (C) Viabilities of U87MG cells with different treatments. (D) Tumor growth curves of mice intratumorally injected with M-SAO@SiO_2_, SAO@SiO_2_, and PBS, followed by X-ray irradiation. M-SAO@SiO_2_: Nanosensitizers consisted of a core made of SrAl_2_O_4_:Eu (SAO) and a silica coating loaded with merocyanine 540 (MC540), SAO@SiO_2_: Silica-coated SrAl_2_O_4_:Eu. Adapted with permission from Ref 8. Copyright 2015 American Chemical Society.

Our group developed an X-PDT strategy based on the type II PDT mechanism and achieved efficient, low-dose* in vivo* cancer treatment 8. The nanosensitizers consisted of a core made of SrAl_2_O_4_:Eu (SAO) and a silica coating loaded with merocyanine 540 (MC540), as shown in Figure 10A. Under X-ray irradiation, SAO emitted bright green luminescence, which was well absorbed by the loaded photosensitizer, MC540 (Figure 10B). ^1^O_2_ was generated following a typical type II PDT mechanism. Compared to the low cytotoxicity of controls, the X-ray irradiation of radioresistant human glioblastoma (U87MG) cells pre-incubated with MC540-loaded mesoporous silica coated SAO (M-SAO@SiO_2_) nanosensitizers showed a viability drop to 38% (Figure 10C). Furthermore, *in vivo* X-PDT studies demonstrated that subcutaneous U87MG tumor growth was almost completely inhibited after an intratumoral injection of the M-SAO@SiO_2_ nanosensitizers, as compared with the controls (Figure 10D). In sum, *in vitro* and *in vivo* studies have shown that a low dose of X-rays (0.5 Gy, single dose) sufficiently damaged radioresistant cancer cells and caused tumors shrinkage, while leaving normal tissue unaffected. Moreover, unlike the previously studied scintillators, SAOs are highly hydrolytic and can be degraded into low-toxic ions and cleared from the host, causing no long-term side effects.

**Figure 11:**
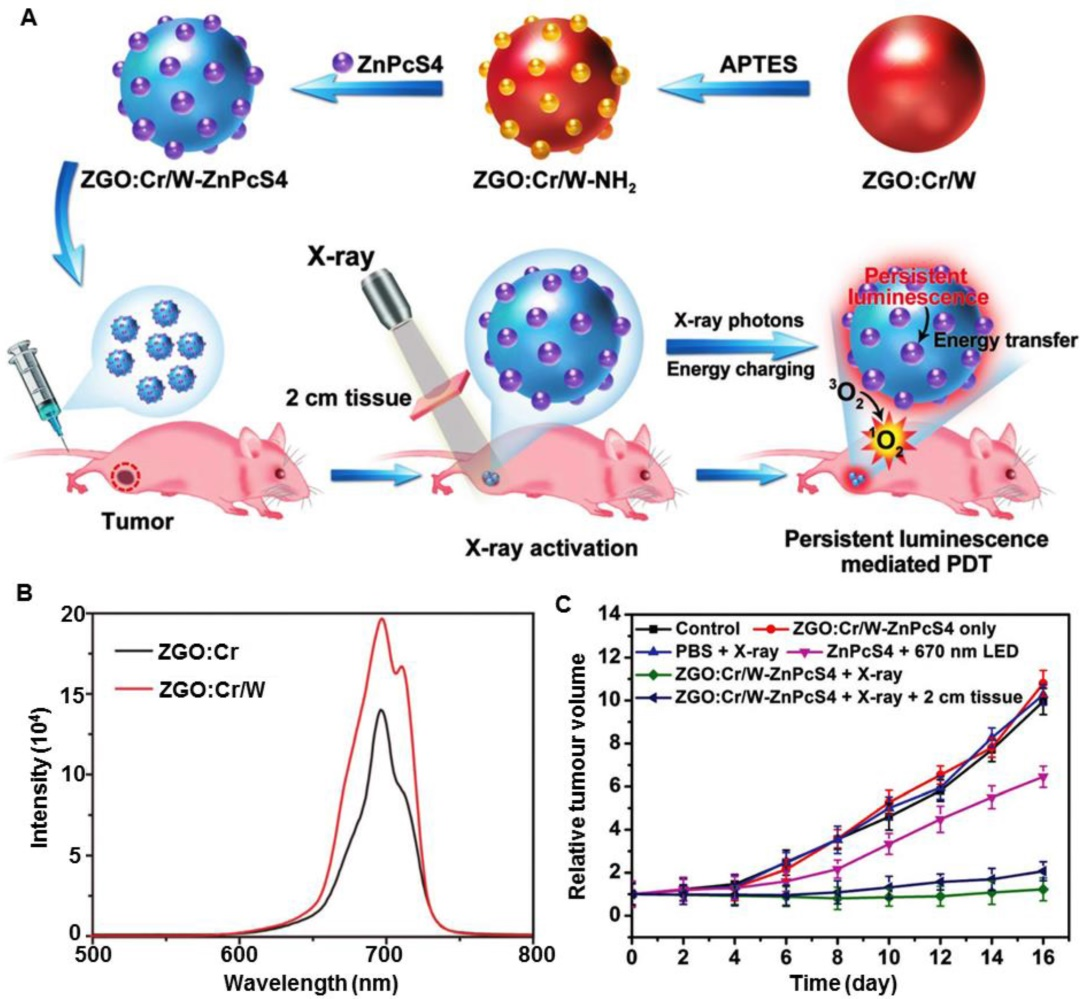
(A) Scheme of the synthesis of ZGO:Cr/W-ZnPcS4 and its performance in the subcutaneous tumor model. (B) X-ray excited persistent luminescence spectra of ZGO:Cr/W and ZGO:Cr nanoparticles. (C) Tumor growth curves for different groups of tumor-bearing mice after various treatments. Adapted with permission from Ref 51. Copyright 2018 WILEY-VCH.

Heavy (high-Z) elements can enhance the absorption capacity of X-ray irradiation and have been used clinically for imaging 28, 50. The enhancement is also helpful for both the PDT and RT steps of X-PDT, which have been demonstrated in recent studies of X-PDT by incorporating heavy atoms into nanosensitizers. For example, the Yang group doped W(VI) into ZnGa_2_O_4_:Cr (ZGO:Cr/W) and achieved ultralow-dose X-ray activation for cancer treatment (Figure 11A) 51. As shown in Figure 11B, after doping with W(VI), the intensity of XEOL increased sharply compared with the nanoscintillators without W(VI). Interestingly, the X-rays excited afterglow luminescence could activate a long-term PDT process. *In vivo* experiments demonstrated that low-dose X-ray irradiation (0.18 Gy) was enough to activate the X-PDT process and cause significant delay of tumor growth (Figure 11C). These advances allow X-PDT to be activated by intermittent X-ray irradiation, which can further minimize ionizing-irradiation induced toxicity. Furthermore, the Li group prepared Gd_2_(WO_4_)_3_:Tb nanoparticles and demonstrated their potential for use in multifunctional theranostics for CT/MRI-guided X-PDT, which showed a higher tumor growth inhibition efficiency at a low X-ray dose (6 Gy) than that seen for RT alone 52.

**Figure 12:**
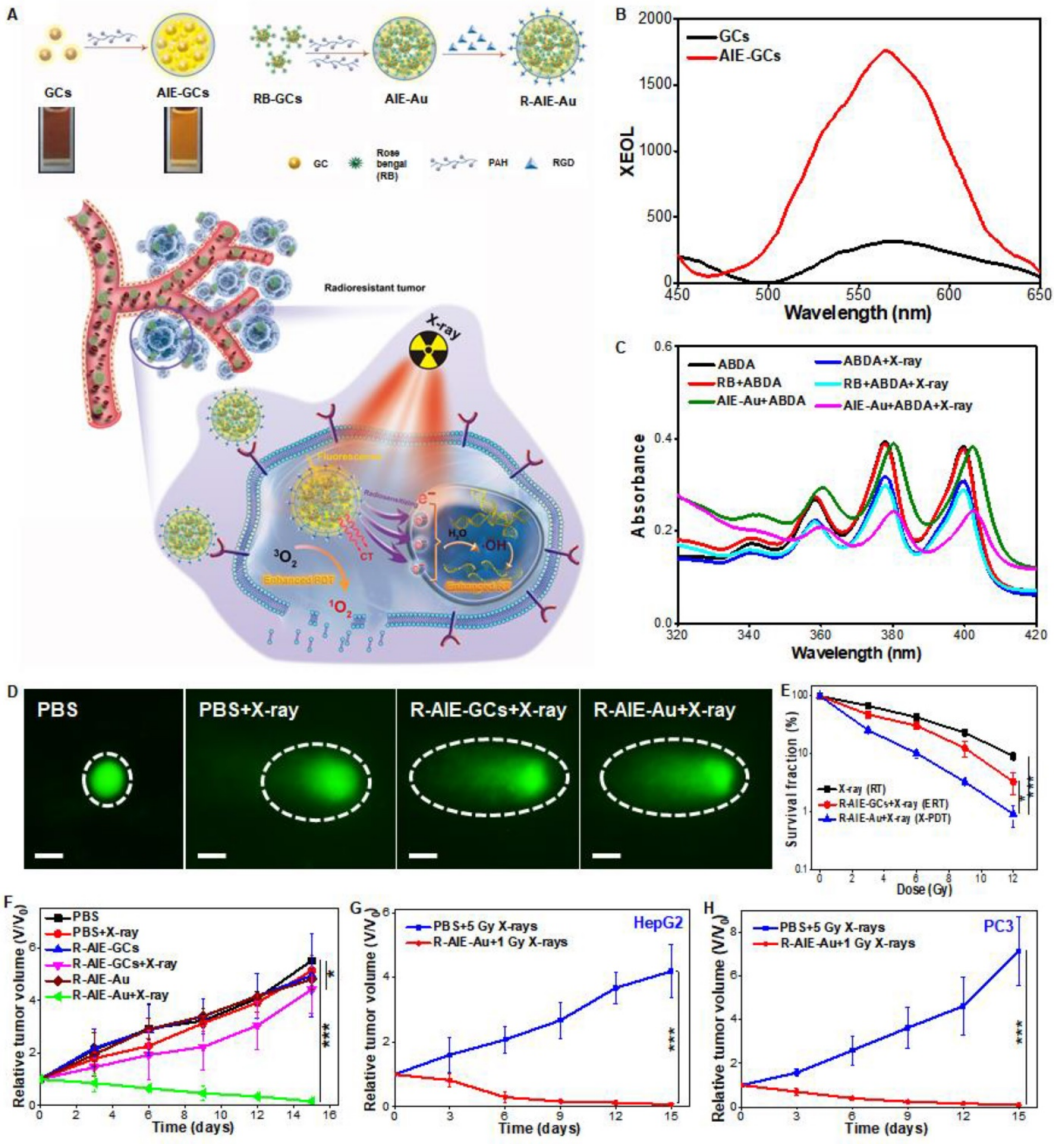
(A) Schematic illustrations of the process of the preparation of silicate nanosensitizers (RGD-ZSM-RB) and of the mechanism of RGD-ZSM-RB mediated X-PDT. (B) The XEOL spectra of GCs and AIE-GCs. (C) The absorption spectra of ABDA (^1^O_2_-detection probe) in different solutions with or without X-ray irradiation (50 kV, 70 μA). (D) DNA damage (dashed circle) assessed by single cell electrophoresis assays (Scale bar, 10 µm). (E) Cell proliferation capacity measured by clonogenic assays. Tumor growth curves of (F) U87MG, (G) HepG2 and (H) PC3 tumor-bearing mice after different treatments. Adapted with permission from Ref 56. Copyright 2019 WILEY-VCH.

Au has great radio-sensitization effect, owing to the strong interaction with ionizing radiation 53. Osakada et al. found bovine serum albumin (BSA) protected Au clusters (Au-BSA) could generate XEOL with an emission peak at 667 nm under hard-X-rays 54. Although these Au clusters only were excited by hard-X-rays 55, the preliminary results proposed the possibility of using biomoleculse (such as BSA) to mediate the activation of X-ray irradiation on metal clusters. Our group reported the aggregates (AIE-Au) of conjugations of glutathione-protected gold clusters (GCs) and rose bengal (RB) (Figure 12A) 56. The AIE-GCs enhanced the X-ray-excited luminescence by 5.2-fold than that of single GCs (Figure 12B). X-ray irradiation could stimulate X-PDT effect in cells (Figure 12C). Meanwhile, an enhanced radiatherapy effect was also activated, due to the large amount of radiosensitive high-Z gold atoms in AIE-Au nanoparticles (Figure 12D). The combined therapy resulted in significantly reduced U87MG cells proliferation (Figure 12E). The experiments *in vivo* showed that AIE-Au could effectively suppress tumor growth by triggering the generation of reactive oxygen species (^1^O_2_ and •OH) under low-dose X-ray irradiation, and realize highly effective treatment of various irradiation-resistant tumors through the unique X-PDT mechanism (Figure 12F-H).

**Figure 13:**
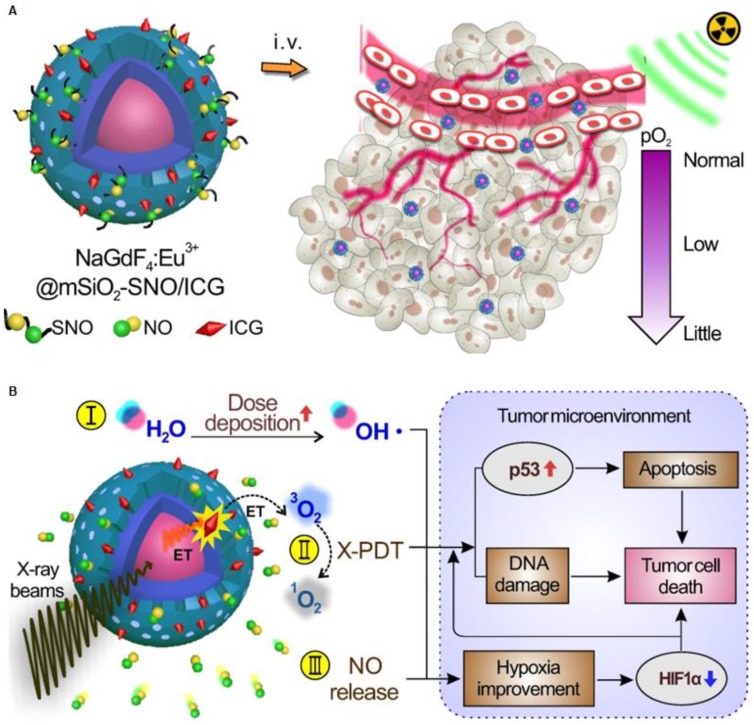
(A) Schematic of the structure of NSC@mSiO2-SNO/ICG NPs and their passive accumulation in tumors via the EPR effect. (B) X-ray radiation on this system would trigger multiple tumoricidal responses by: (I) increasing dose deposition to accelerate radiolysis, (II) producing cytotoxic ROS by activating ICG based on the scintillation effect of NSC, and (III) releasing high levels of NO due to radiation fracture of S-N bonds. Adapted with permission from Ref 57. Copyright 2018 Ivyspring Publisher.

Moreover, Dou et al. prepared a radiation-responsive scintillating nanotheranostic system (NSC@mSiO_2_-SNO/ICG) by loading S-nitrosothiol groups (SNO, a NO donor) and indocyanine green (ICG, a photosensitizer) on mesoporous silica-coated Eu^3+^-doped NaGdF_4_ scintillating nanocrystals (NSC) (Figure 13A) 57. The energy transfered from NSC to ICG activated the X-PDT process to generate large amount of ROS. Meanwhile, the S-N bond in SNO was excited to produce high NO concentration in tumors under X-ray irradiation, which improves the tumor hypoxia due to the enhanced vasodilation. (Figure 13B).

**Figure 14:**
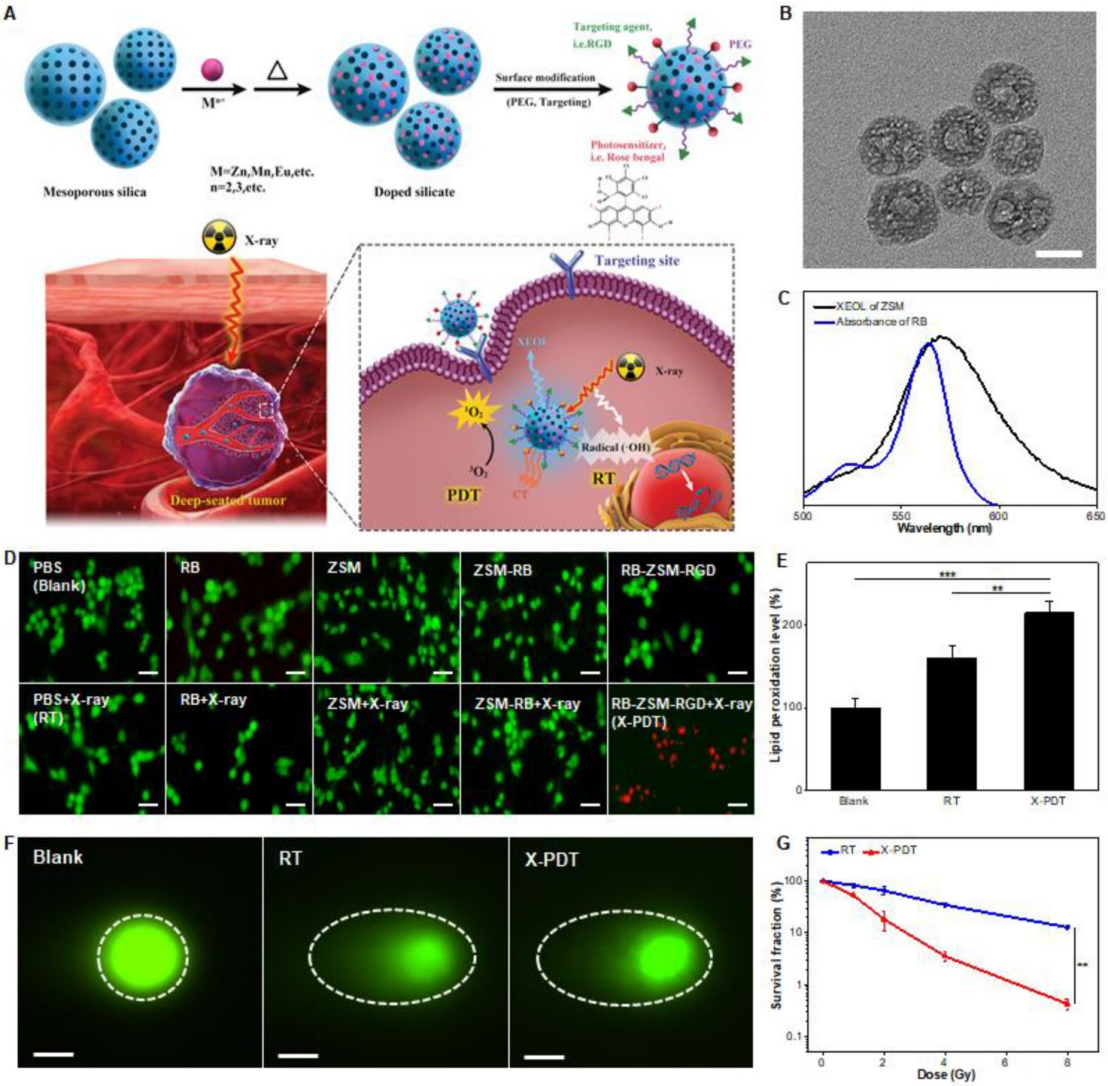
(A) Schematic illustrations of the process of the preparation of silicate nanosensitizers (RGD-ZSM-RB) and of the mechanism of RGD-ZSM-RB mediated X-PDT. (B) TEM image of silicate nanosensitizers (Scale bar, 50 nm). (C) The overlap of the XEOL spectrum of ZSM and the absorption spectrum of RB. (D) Fluorescence images of calcein AM (green fluorescence for live cells) and PI (red fluorescence for dead cells) co-stained U87MG cells with different treatments (Scale bar, 100 µm). (E) Lipid damage assessment measured by lipid peroxidation assays. (F) DNA damage (dashed circle) assessed by single cell electrophoresis assays (Scale bar, 25 µm). (G) Cell reproductive capacity measured by clonogenic assays taken 10 d after RT or X-PDT. Adapted with permission from Ref 58. Copyright 2019 WILEY-VCH.

As opposed to incorporating high-Z elements into the matrix of scintillators, bioconjugation of clinical photosensitizers containing high-Z elements would be a better choice to increase the cell cytotoxicity and minimize the side effects. We employed a mesoporous silica-templating to prepare uniform and monodisperse silicates (Figure 14A,B) 58. By simply adjusting the metal dopants, silicate nanoscintillators with a controlled size and X-ray excited optical luminescence (450-900 nm) were synthesized. They were than modified with rose bengal (RB), a photosensitizer with four iodine atoms and a drug commonly used in clinical trials (Clinical Trials ID NCT02288897). Typically, silicates with Zn- and Mn-dopants (ZSM) emitted XEOL that matched well with the absorption of RB (Figure 14C). Under a low-dose of X-ray irradiation (0.75 Gy), the silicate nanosensitizers achieved excellent reactive oxygen species (ROS) generation and significant inhibitory effects on tumor progression were observed, while minimally affecting normal tissues (Figure 14A). X-ray energy can be deposited in the nanoscintillators to generate the PDT process, inducing cell death (Figure 14D,E). The addition of iodine to radiosensitizers can be utilized to enhance the RT efficiency by increasing both the yield and the effects of •OH (Figure 14F), thereby enhancing the efficacy of X-PDT (Figure 14G).

**Figure 15:**
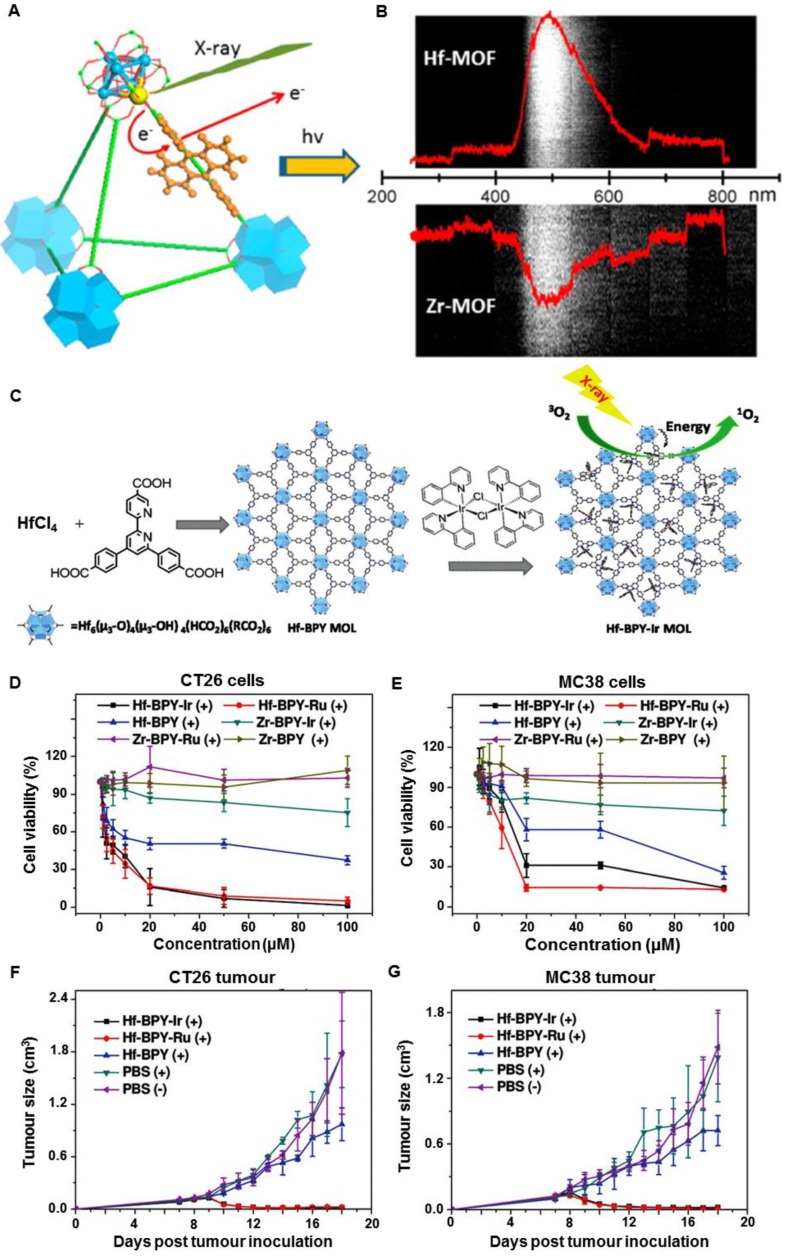
(A) Scheme showing X-ray induced generation of fast photoelectrons from heavy metals followed by scintillation of the anthracene-based linkers in the visible spectrum. (B) Optical spectra of Hf-MOFs and Zr-MOFs induced by X-ray irradiation at a dose of 6 Gy·min^-1^. Adapted with permission from Ref 59. Copyright 2014 American Chemical Society. (C) Schematic illustration of the synthesis of Hf-based MOLs and MOL-enabled X-PDT to generate ^1^O_2_. Viabilities of (D) CT26 cells and (E) MC38 cells with different treatments. (F) CT26 and (G) MC38 tumor growth curves after different treatments. Adapted with permission from Ref 9. Copyright 2017 Wiley-VCH.

Hafnium oxide has been clinically used to enhance RT in both Europe and the US (https://www.nanobiotix.com/_en/news). In 2004, the Lin group found that heavy metal clusters and luminescent organic bridging ligands could be synergistically assembled in metal-organic frameworks (MOFs) to achieve efficient X-ray scintillation (Figure 15A) 59. The MOFs employed high-Z metal clusters M_6_(μ_3_-O)_4_(μ_3_-OH)_4_(carboxylate)_12_ (M = Hf or Zr) as connecting nodes and an anthracene-based emitter as the bridging ligand. Upon irradiation of X-rays (20-200 keV), the MOFs emitted XEOL in the visible light region (Figure 15B). In 2017, the Lin group reported metal-organic layers (MOLs) that were built from [Hf_6_O_4_(OH)_4_(HCO_2_)_6_] and Ir[bpy(ppy)_2_]^+^ or [Ru(bpy)_3_]^2+^ ligands (Figure 15C) 9. Both ligands were efficient photosensitizers, with high quantum yields of ^1^O_2_. The Ir-based MOLs generated ^1^O_2_ more efficiently and induced more cancer cell death than the Ru-based MOLs upon X-ray irradiation. In addition, Hf-MOLs had a much higher ^1^O_2_ generation efficiency than Zr-MOLs, which could be attributed to the better absorption of X-ray energy by the heavier Hf atom compared to the Zr atom. The *in vitro* and *in vivo* studies indicated that cancer cells were effectively killed and tumor regression was observed on CT26 and MC38 tumor models after an intratumoral injection of Hf-MOLs followed by X-ray irradiation (Figure 15D-G). These discoveries confirmed that MOFs containing heavy metal secondary building units displayed superb RT efficiency in these tumor models. Furthermore, the Lin group optimized the structural and compositional tunability of MOFs based on electron-dense Hf_12_ or Hf_6_ clusters and strongly photosensitizing Ir(DBB)[dF(CF_3_)ppy]^2+^ bridging ligands to improve the efficacy of X-PDT 60. Upon X-ray irradiation, the Hf_12_ or Hf_6_ clusters efficiently absorbed X-rays to enhance RT by producing •OH and induced the PDT process through the excitation of Ir(bpy)[dF(CF_3_)ppy]^2+^ derived ligands to generate ^1^O_2_ and superoxide anions. *In vitro* and *in vivo* experiments showed that Hf_12_-Ir and Hf_6_-Ir MOFs promoted effective cell death by combining RT and PDT, resulting in significant tumor regression at low X-ray doses (0.5 × 5 Gy).

Selective delivery of photosensitizers to the mitochondria of cancer cells can enhance the therapeutic efficacy. To achieve this, the Lin group prepared Hf-DBB-Ru (DBB-Ru = bis(2,2'-bipyridine)(5,5'-di(4-benzoato)-2,2'-bipyridine)ruthenium(II) chloride) as a mitochondria-targeted MOF for X-PDT 61. By incorporating cationic Ru-based photosensitizers, the cationic MOFs were successfully constructed. The cationic MOFs exhibited strong mitochondria-targeting properties. Upon X-ray irradiation, Hf-DBB-Ru efficiently generated •OH from the Hf_6_ clusters and ^1^O_2_ from the DBB-Ru photosensitizers to produce an X-PDT effect. *In vivo* studies demonstrated that the mitochondria-targeted X-PDT depolarized the mitochondrial membrane to initiate apoptosis of cancer cells, leading to significant regression of colorectal tumors in mouse models.

**Figure 16:**
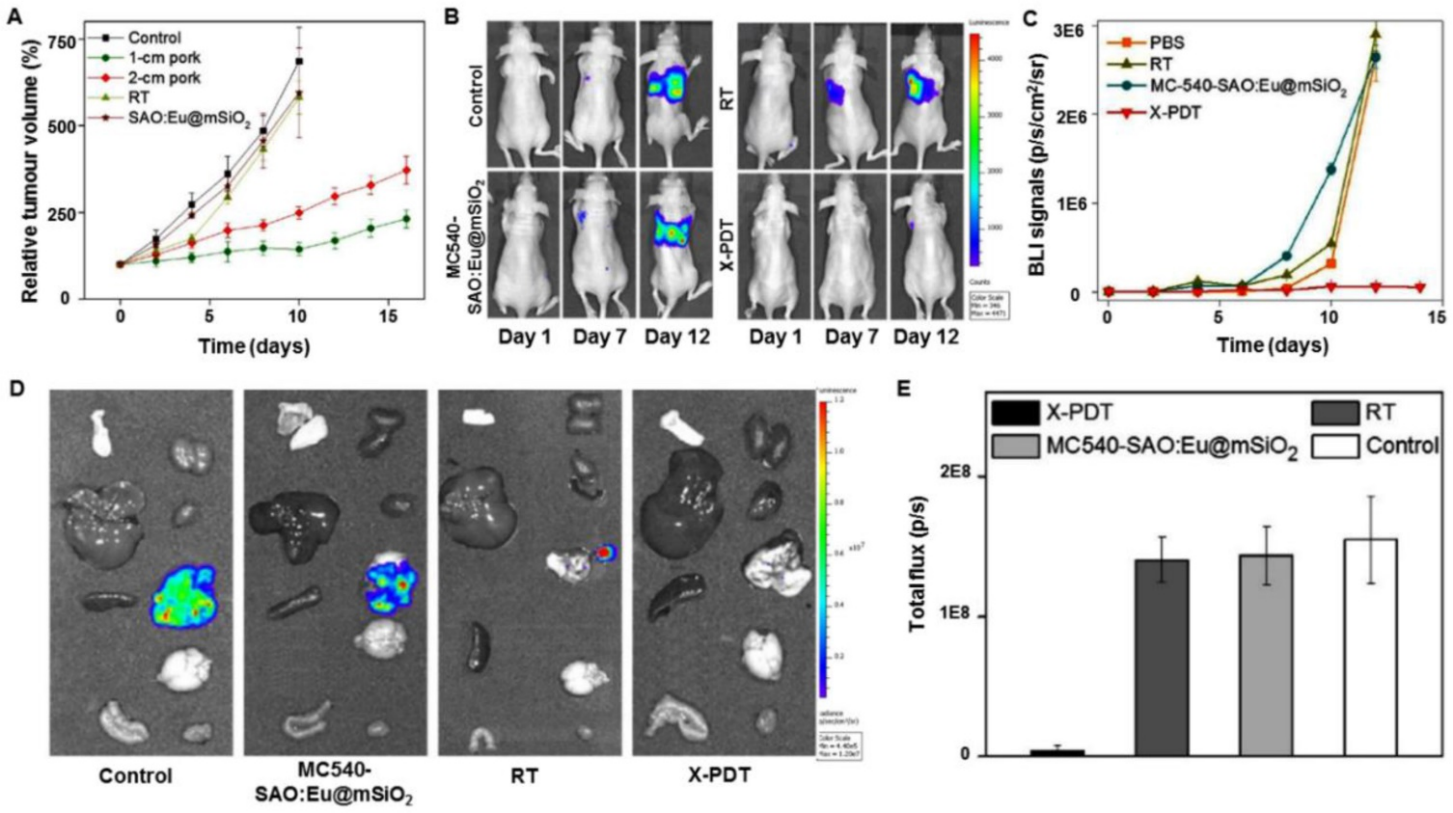
(A) X-PDT efficacy to treat subcutaneously implanted tumors from above thick tissue. (B) Representative bioluminescence images of mice that were injected with a SAO:Eu nanosensitizers and firefly luciferase expressing H1299 cell mixture to the thorax and treated by X-PDT, RT, and Controls. (C) Tumor growth measured by monitoring the bioluminescence imaging (BLI) signal changes at different time points. (D) *Ex vivo* bioluminescence images taken immediately after tissue dissection. The organs were organized in the following order: left column (from top to bottom): skin, liver, spleen and intestine; right column (from top to bottom): kidneys, heart, lung, brain and muscle. (E) BLI signals from the lungs based on the ROI analyses on (B). Adapted with permission from Ref 16. Copyright 2016 Ivyspring Publisher.

To advance this technology, it is critical to demonstrate that nanoparticles can be injected systemically and, following external X-ray irradiation, activate X-PDT *in situ* to kill cancer cells in deep tissues. For this purpose, it is paramount to investigate the treatment in a clinically relevant model, not a subcutaneous tumor model. To simulate a clinically relevant model, deep-seated tumors were mimicked by putting a layer of pork (1-2 cm thickness) between a subcutaneous lung tumor model and an X-ray generator, and the efficacy of X-PDT was evaluated 16. After injecting SAO:Eu@mSiO_2_ intratumorally and irradiating with X-rays, tumor growth was slowed in all the treatment groups, while tumors in the control group grew very rapidly (Figure 16A). The results suggested that tissue thickness had little effect on the efficacy of X-PDT. Encouraged by the excellent tumor growth inhibition, the X-PDT efficacy was then investigated by injecting the mixture of MC540-SAO:Eu@mSiO_2_ nanosensitizers and firefly luciferase expressing H1299 cells into the thorax of mice. Radiation (5 Gy) was applied to the tumor inoculation sites, and the tumor growth was then monitored *in vivo* by bioluminescence imaging (BLI). In X-PDT treated animals, the BLI signals were significantly suppressed, and in other groups, the BLI signals were detected in the lung areas on day 7, with the signals increasing further afterwards (Figure 16B,C). *Ex vivo* imaging confirmed the efficacy of X-PDT induced tumor suppression, finding strong residual signals in the lungs of control animals but close-to-background signals from the X-PDT group (Figure 16D,E).

**Figure 17:**
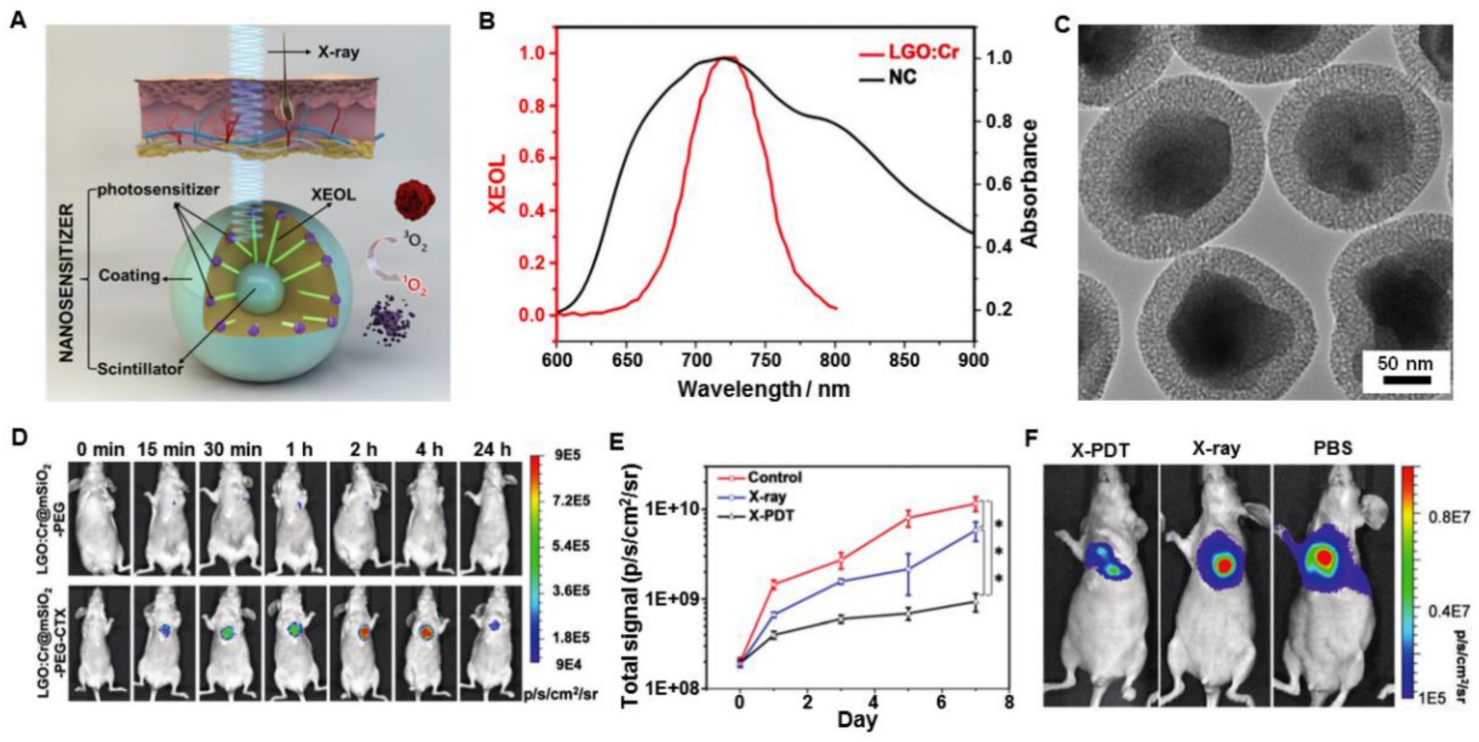
(A) Schematic illustration for the design and mechanism of LGO:Cr@mSiO_2_ mediated X-PDT. (B) The absorption spectrum of 2,3-naphthalocyanine (NC) (black) and the XEOL spectrum of LGO:Cr (red). (C) TEM image of LGO:Cr@mSiO_2_. (D) *In vivo* imaging based on X-ray excited persistent luminescence signals and assessed in H1299 lung tumor models. (E) Tumor growth assessed by monitoring BLI signal changes at different time points. (F) Representative bioluminescence imaging for the three treatment groups taken on day 7. Adapted with permission from Ref 62. Copyright 2017 Royal Society of Chemistry.

It is critical to find a way to track the particle migration *in vivo* and navigate external irradiation to tumor areas. These questions have not yet been addressed and the considerations are different from *in vitro* studies with regard to materials, nanoparticle engineering, experimental designs, and toxicity. For the limited number of *in vivo* studies described so far, nanoparticles were all injected directly into tumors. To this end, one question which needed to be answered was how to navigate the treatment of tumors with accuracy using external irradiation. This issue was resolved by using LiGa_5_O_8_:Cr (LGO:Cr)-based nanoparticles as transducers for imaging-guided *in vivo* X-PDT (Figure 17A) 62. LGO:Cr emitted persistent, near-infrared X-ray luminescence (Figure 17B), which was then coated with a layer of mesoporous silica to match the photosensitizers (2,3-naphthalocyanine (NC)) and modify the surface (Figure 17C). Specifically, after PEGylation and conjugation with the targeting agent cetuximab (CTX), the LGO:Cr@mSiO_2_-PEG-CTX nanosensitizers were able to accumulate efficiently in the H1299 orthotopic non-small cell lung cancer tumors implanted into the lung after intravenous injection and were confirmed by monitoring the X-ray luminescence from LGO:Cr (Figure 17D). The tumor selectivity was further improved by navigating the X-ray irradiation to the tumor areas with imaging guidance. Guided by the imaging, external irradiation was applied, leading to efficient tumor suppression while minimally affecting normal tissue (Figure 17E). At the end of the therapy, the X-PDT group showed lower BLI signals than that of control groups, further confirming the therapeutic efficacy (Figure 17F).

**Figure 18:**
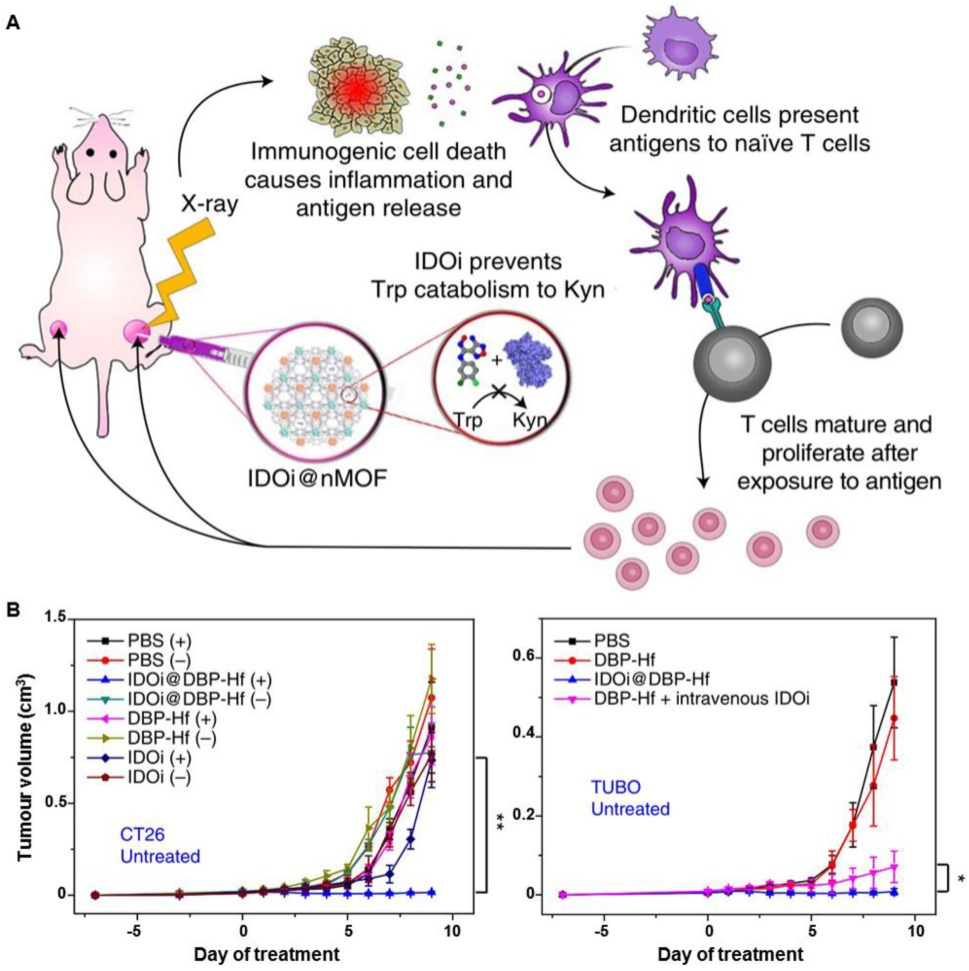
(A) Scheme of nano-MOF enabled synergistic X-RDT and immunotherapy using extremely low doses of X-rays. (B) Growth curves of CT26 and TUBO tumor-bearing mice intravenously administered with PBS, DBP-Hf, IDOi@DBP-Hf, or DBP-Hf + IDOi, with or without X-ray irradiation. Adapted with permission from Ref 7. Copyright 2018 Nature Publishing Group.

As a traditional clinical therapy methodology, RT has become a very important strategy for cancer management. In recent years, immunotherapy has been of great interest to researchers, clinicians, and pharmaceutical companies. New strategies for the combination of traditional RT and emerging immunotherapy for cancer treatment have been developed in parallel. The Lin group have recently confirmed a synergistic effect when combining nano-MOFs with cancer immunotherapy 7. In that research, the Lin group bridged Hf/Zr-MOFs-enabled X-PDT and checkpoint blockade immunotherapy to develop radio-enhancers for X-ray radiotherapy for both local and systemic tumor elimination (Figure 18A). In all radioresistant head and neck squamous cell carcinoma (SQ20B), glioblastoma (U87MG), prostate cancer (PC-3), and colorectal cancer (CT26) tumor models, intratumoral delivery of 5,15-di(*p*-benzoato)porphyrin (DBP)-Hf and 5,10,15,20-tetra(*p*-benzoato)porphyrin (TBP)-Hf nano-MOFs caused efficient tumor regression at low doses of X-ray irradiation. When loaded with an inhibitor of the immune checkpoint molecule indoleamine 2,3-dioxygenase (IDOi), consistent abscopal responses were observed in all treatment of CT26 and TUBO tumor models with low-doses of X-rays (Figure 18B). The treatment not only minimized the side effects of local RT, but also caused systemic immunity to efficiently inhibit tumor growth. By combining the advantages of local RT and systemic tumor rejection *via* synergistic X-ray-induced *in situ* vaccination and indoleamine 2,3-dioxygenase inhibition, MOF-based nanoplatforms may overcome some of the limitations of RT and checkpoint blockades in cancer treatment. Based on this radio-immuno metal-organic (RiMO) technology, a Phase 1 study of RiMO-301 in patients with advanced tumors has been initiated (https://clinicaltrials.gov/ct2/show/NCT03444714).

## Future perspectives

Although X-PDT has demonstrated good efficacy and benefits, the development of this new therapeutic method is still in its infancy. As discussed above, X-PDT treatment is essentially a combination therapy of PDT and ionizing irradiation. However, how to deal with the interplay between the two methods and how we can further improve this synergy by tuning irradiation parameters and/or changing a transducer's targeting ability remain a major challenge.

By now, only two simulations have been proposed. These simulations were the photon cross section model and the electron cross section model. Both simulations used fluorides as a case study and concluded that an irradiation level in excess of 60 Gy was required to achieve enough ^1^O_2_ per cell to deliver a killing dose. *In vivo* investigations on xenograft tumor models (both of subcutaneous and orthotopic tumors) demonstrated that low-dosages of X-rays (<5 Gy) were sufficient to inhibit or eradicate tumor growth by both intratumoral injection and systemic administration. These *in vivo* experimental results were inconsistent with the conclusions from the simulations. So, perhaps, there are additional mechanisms besides the PDT and RT processes at work. More investigation into the contribution of X-ray luminescence and fluorescence on cell lethality should be performed. UV and other ionizing irradiation was generated during the scintillation process. Deep-UV light alone can be harnessed to damage cells and cancerous tissues. In sum, it is fair to admit that X-PDT is intrinsically a complicated process and cell death is the result of multiple factors. These understudied factors could account for the discrepancy between the theoretical models and experimental studies. So, the mechanism by which X-PDT induces cell death should be carefully investigated based on the experimental results.

It is important to note that exploiting methods to improve the energy conversion and safety profiles of scintillators is key to X-PDT. There are several aspects to consider when engineering scintillators including: (1) constructing scintillators that have a large X-ray absorption cross-section, high conversion efficiency of X-rays to visible photons, and optimized spatial positioning of the molecular entities involved; (2) reducing the overall size of the transducers, which must be balanced against the loss of energy conversion efficiency. It is noteworthy that many of the reported transducers in X-PDT have a relatively large size, which is suboptimal for tumor targeting; and (3) striking a balance between short-term stability and fast biodegradation of scintillators. The high XEOL efficiency of transducers under low-dose X-rays should be initially considered. Moreover, the transducers with a controlled size distribution in the range of 50-200 nm should exhibit prolonged blood circulation, a relatively low rate of uptake by the reticuloendothelial or mononuclear phagocyte systems, and an increased rate of tumoral uptake based on enhanced permeability and retention effects. Additionally, the modification of stealth components (*e.g.*, polyethylene glycol, zwitterionic molecules) onto the surface of nanoparticles can reduce nonspecific protein corona formation *in vivo*. Most importantly, the nanosensitizers should be non-toxic and easily metabolized with low long-term toxicity. Most of the excellent scintillator candidates, such as aluminate and all-inorganic perovskites, are hydrolytic in nature, and could quickly break up into their constituent ions when exposed to water. One solution to this problem is to use coating materials to decorate hydrolytic scintillator cores so as to enhance their physiological stability in media. The materials/ coating strategies should be exploited to modulate the stability and degradation of scintillators *in vivo*. Moreover, the number of photosensitizers loaded in scintillators and the distance between them determine the energy transfer efficiency from scintillators to photosensitizers. Therefore, a covalent conjugation strategy for combining photosensitizers and scintillators with controllable intra-component distance may be more applicable than a physical loading method. So far, there have been few studies that systematically examine these on X-PDT efficacy.

So far, X-PDT has been demonstrated mostly *in vitro* or with subcutaneous tumor models *in vivo*. It is expected that many clinically relevant tumor models, especially the deeply-located tumors, could be cured by this methodology. Besides, most of the *in vivo* studies are conducted by intratumoral injection of nanosensitizers, which is not conducive to the non-invasive clinical treatment of deep orthotopic cancer. This would call for great specificity in the uptake of the X-PDT nanoplatforms in these cancer cells. It could be completed by introducing *in vivo* active targeting effects through conjugation with various cancer targeting agents, such as folic acid, RGD (for integrin α_v_β_3_), cetuximab and panitumumab (for epidermal growth factor receptor), herceptin (for human epidermal growth factor receptor 2), bevacizumab (for vascular endothelial growth factor) and so on. For example, mitochondria-targeting agents achieved high accumulation in mitochondria, and X-ray irradiation produced ROS which induced significantly mitochondrial collapse and cellular apoptosis than X-ray alone. So, the methodology of altering the energy deposition profile in cells may contribute to the cell killing mechanism of X-PDT.

The low-dose irradiation activated X-PDT holds the potential for clinical translation as an alternative to ionizing irradiation therapy. It is important to compare the two methods in a clinical environment to assess the benefits and drawbacks of X-PDT with regards to therapeutic efficacy and side effects. It is meaningful to evaluate the capacity of X-PDT to treat radiation-refractory tumors. In RT, pre-treatment functional imaging (*e.g.*, positron emission tomography) is often performed to stage tumors and guide irradiation planning. However, functional imaging is not permitted in an irradiation room, and a change in the status of the patient from pre-scans may occur, leading to setup errors. Many scintillators contain high-Z elements, making them visible under on-board CT. It is thus possible to use these scintillators to not only regulate PDT, but also to guide the irradiation to minimize damage to normal tissue. These possibilities should also be investigated to facilitate clinical translation of X-PDT.

## Conclusions

There have been dramatic developments in the feasibility of X-PDT as a novel cancer treatment methodology over the past decades. X-PDT has shown promising therapeutic effects by combining PDT and RT methods to treat deep-seated tumors. In this review, we have attempted to provide an overview of the research developments in X-PDT strategy, including the concept, the related design parameters, the combined therapy mechanism, the biomedical applications, and the concluding prospects. As a synergistic PDT and RT procedure, X-ray-activated PDT overcomes the limitation of light penetration depth in traditional light-activated PDT, and less irradiation is needed in X-PDT for tumor ablation than in traditional RT. However, the real biomedical applications of X-PDT are still in the early development stage. We hope that this review will provide a timely overview of the current situation in this field and point to new positive directions in the battle against cancer.

## Figures and Tables

**Table 1:** Investigations of X-PDT.

Major milestones	Publication Year	Dose	Transducer	Size /nm	Photosensitizers	Attachment strategy	Exp. subject	Ref.
Concept proposed	2006	N/A	LaF_3_:Ce, LuF_3_:Ce, CaF_2_:Mn, CaF_2_:Eu, BaFBr:Eu, BaFBr:Mn, CaPO_4_:Mn ZnO, ZnS, TiO_2_	N/A	N/A	N/A	concept	10
								
Simulation	2009	0.1-100 Gy	LaF_3_:Ce, LuF_3_:Ce, CaF_2_:Mn, CaF_2_:Eu, BaFBr:Eu, BaFBr:Mn, CaPO_4_:Mn ZnO, ZnS, TiO_2_	N/A	photofrin, fullerenes, TiO_2_	N/A	in solution	19
	2015	100/500 keV	Gd_2_O_3_, Gd_2_O_2_S, Lu_2_O_3_, CdSe, InP	100	N/A	N/A	in solution	22
								
Mechanistic study	2016	5 Gy	SrAl_2_O_4_:Eu@mSiO_2_	73.5	MC540	pore loading	animal (it)	16
								
Solution evaluation	2008	250 kV, 0.44 Gy·min^-1^	LaF_3_:Tb	15	MTCP	covalent binding	in solution	33
	2015	75 kV, 20 mA	LaF_3_:Tb	39	rose bengal	covalent binding	in solution	34
	2015	75 kV, 20 mA	LaF_3_:Tb @SiO_2_	40	rose bengal	covalent binding	in solution	35
	2013	44 kV, 40 mA, 14.6 Gy	Tb_2_O_3_	3	porphyrin	covalent binding	in solution	36
	2015	400 mA, 10^15^ photons·s^-1^	GdEuC12 micelles	4.6	hypericin	physical loading	in solution	37
	2016	40 kV, 15 mA	[M_6_L^i^_8_L^a^_6_]^n^ complexes	N/A	self	complex	in solution	39
								
*In vitro* evaluation	2007	1-10 Gy	TiO_2_, ZnS:Ag, CeF_3_, CdTe and CdSe	N/A	self	N/A	HeLa cells	40
	2011	120 kVp, 20 mA	Gd_2_O_2_S:Tb	20 μm	photofrin II	co-location	glioblastoma cells	41
	2011	2 Gy	Y_2_O_3_	12	psoralen	physical attachment	PC3 cells	42
	2014	120 kV, 2 Gy	ZnS:Cu,Co	4	TBrRh123	covalent binding	PC3 cells	45
	2015	6 MV, 2 Gy	SiC/SiO_x_ nanowires	20	H_2_TPACPP	covalent binding	A549 cells	46
								
*In vivo* evaluation (intratumor)	2015	220 keV, 8 Gy	LiYF_4_:Ce	40	ZnO	coating	animal (it)	49
	2018	80 kV, 4 Gy	LiLuF_4_:Ce	30	Ag_3_PO_4_-Pt(IV)	coordination	animal (it)	17
	2017	120 kV, 20 mA	[Hf_6_O_4_(OH)_4_(HCO_2_)_6_] SBUs	500	Ir[bpy ppy)_2_]^+^ [Ru(bpy)_3_]^2+^	post-synthetic metalation	animal (it)	9
	2018	5 × 0.5 Gy	Hf_6_ SBUs, Hf_12_ SBUs	295.3, 91.3	Ir(DBB)[dF(CF_3_)ppy]_2_^+^	post-synthetic metalation	animal (it)	60
	2015	50 kV, 70 μA, 0.5 Gy	SrAl_2_O_4_:Eu	150	MC540	pore loading	animal (it)	8
								
*In vivo* evaluation (intravenous)	2018	50 kV, 60 µA, 0.18 Gy	ZnGa_2_O_4_:Cr/W	15	ZnPcS_4_	pore loading	animal (iv)	51
	2018	250 kVp, 15 mA	Hf-DBB-Ru	98	DBB-Ru	coordination	animal (iv)	61
	2019	50 kV, 70 μA, 6 Gy	Gd_2_(WO_4_)_3_:Tb	50	MC540	physical attachment	animal (iv)	52
	2019	50 kV, 70 μA, 1 Gy	AIE-Au	68.2	rose bengal	bioconjugation	animal (iv)	56
	2019	50 kV, 70 μA, 1 Gy	Zn_2_SiO_4_:Mn	30-120	rose bengal	bioconjugation	animal (iv)	58
								
*In vivo* evaluation (orthotopic tumor)	2017	50 kV, 70 μA, 5 Gy	LiGa_5_O_8_:Cr	100	NC	pore loading	animal (iv)	62
								
Combined therapy	2018	0.5 Gy·fraction^-1^	DBP-Hf, TBP-Hf nMOFs	72	self	post-synthetic metalation	animal (it, iv)	7
